# A missense allele of *KARRIKIN-INSENSITIVE2* impairs ligand-binding and downstream signaling in *Arabidopsis thaliana*

**DOI:** 10.1093/jxb/ery164

**Published:** 2018-05-02

**Authors:** Inhye Lee, Kuglae Kim, Sumin Lee, Seungjun Lee, Eunjin Hwang, Kihye Shin, Dayoung Kim, Jungki Choi, Hyunmo Choi, Jeong Seok Cha, Hoyoung Kim, Rin-A Lee, Suyeong Jeong, Jeongsik Kim, Yumi Kim, Hong Gil Nam, Soon-Ki Park, Hyun-Soo Cho, Moon-Soo Soh

**Affiliations:** 1 Division of Integrative Bioscience and Biotechnology, School of Life Science, Sejong University, Seoul, Republic of Korea; 2 Department of Systems Biology, College of Life Science and Biotechnology, Yonsei University, Seoul, Republic of Korea; 3 Center for Plant Aging Research, Institute for Basic Science (IBS), Daegu, Republic of Korea; 4 Department of New Biology, DGIST, Daegu, Republic of Korea; 5 School of Applied Biosciences, Kyungpook National University, Daegu, Republic of Korea

**Keywords:** Arabidopsis, germination, KAI2, karrikin, photomorphogenesis, phytochrome

## Abstract

A smoke-derived compound, karrikin (KAR), and an endogenous but as yet unidentified KARRIKIN INSENSITIVE2 (KAI2) ligand (KL) have been identified as chemical cues in higher plants that impact on multiple aspects of growth and development. Genetic screening of light-signaling mutants in *Arabidopsis thaliana* has identified a mutant designated as *ply2* (*pleiotropic long hypocotyl2*) that has pleiotropic light-response defects. In this study, we used positional cloning to identify the molecular lesion of *ply2* as a missense mutation of *KAI2*/*HYPOSENSITIVE TO LIGHT*, which causes a single amino acid substitution, Ala219Val. Physiological analysis and genetic epistasis analysis with the KL-signaling components *MORE AXILLARY GROWTH2* (*MAX2*) and *SUPPRESSOR OF MAX2 1* suggested that the pleiotropic phenotypes of the *ply2* mutant can be ascribed to a defect in KL-signaling. Molecular and biochemical analyses revealed that the mutant KAI2^ply2^ protein is impaired in its ligand-binding activity. In support of this conclusion, X-ray crystallography studies suggested that the *KAI2*^*ply2*^ mutation not only results in a narrowed entrance gate for the ligand but also alters the structural flexibility of the helical lid domains. We discuss the structural implications of the Ala219 residue with regard to ligand-specific binding and signaling of KAI2, together with potential functions of KL-signaling in the context of the light-regulatory network in *Arabidopsis thaliana*.

## Teaser

A missense mutation of *KARRIKIN-INSENSITIVE2*, *KAI2*^*ply2*^, compromises its ligand-binding activity, which subsequently impairs KAI2-signaling and multiple aspects of light-dependent responses.

## Introduction

Smoke has long been recognized to contain chemical cues that impact on plant growth and development, and several phytoactive smoke compounds have been identified (Flematti *et al.*, 2004, 2013; van Staden *et al.*, 2004). Among them, the best characterized are karrikins (KARs), a group of butenolides. In plants, responses to KARs include enhancement of seed germination, potentiation of light-responsive seedling establishment, and de-etiolation (Chiwocha *et al.*, 2009; Nelson *et al.*, 2009, 2010). Molecular analysis has shown that KAR-dependent responses of plants entail transcriptional regulation of over one hundred of genes, which include primary KAR-target genes such as *D14-LIKE2* (*DLK2*), *KAR-UP F-BOX1* (*KUF1*), and *SALT TOLERANCE HOMOLOG7* (*STH7*) (Nelson *et al.*, 2009, 2010).

Recent studies have shown that plants are equipped with a smoke sensory system for karrikin (Morffy *et al.*, 2016). A few components involved in KAR-signaling have been identified in *Arabidopsis thaliana*, including an α/β hydrolase fold protein, KARRIKIN INSENSITIVE2/HYPOSENSITIVE TO LIGHT (hereafter referred to as KAI2), an F-box protein, MORE AXILLARY GROWTH2 (MAX2, also referred to as ORESARA9/PLEIOTROPIC PHOTO SIGNALING/KARRIKIN INSENSITIVE1), and SUPPRESSOR OF MAX2 1 (SMAX1)/SMAX1-LIKE2 (SMXL2) (Nelson *et al.*, 2011; Sun and Ni, 2011; Waters *et al.*, 2012; Stanga *et al.*, 2013, 2016). Crystallographic studies and biochemical analyses have demonstrated the role of KAI2 as a KAR receptor (Bythell-Douglas *et al.*, 2013; Guo *et al.*, 2013; Kagiyama *et al.*, 2013; Zhao *et al.*, 2013; Toh *et al.*, 2014). By analogy to the well-defined mode of action of strigolactone (SL)-signaling, in which MAX2 forms a co-receptor with AtDWARF14 (AtD14), a paralog of KAI2, to target SMXLs/DWARF53 for ubiquitin-mediated degradation (Jiang *et al.*, 2013; Zhou *et al.*, 2013; Soundappan *et al.*, 2015; Yao *et al.*, 2016), it has been postulated that the KAI2–MAX2 complex would lead to ligand-dependent degradation of SMAX1/SMXL2, thus relieving a repressive gene regulation (Morffy *et al.*, 2016; De Cuyper *et al.*, 2017). However, KAI2 signaling may involve distinct biochemical mechanisms. For example, SL perception targets its receptor, AtD14, for MAX2-dependent ubiquitin-mediated degradation (Chevalier *et al.*, 2014; Zhao *et al.*, 2015), whereas KAR induces degradation of KAI2, independently of MAX2, via a non-proteosomal pathway as yet unknown (Waters *et al.*, 2015*a*). In addition, it remains unclear how SMAX1/SMXL2 specifically repress the KAR-dependent responses whilst not affecting SL-dependent responses. Thus, the detailed mode of action of KAR-signaling remains to be elucidated.

Phylogenomic analyses combined with cross-species complementation assays using KAR- or SL-receptor mutants have not only dissected the biological impact of KAR but also raised an intriguing hypothesis, namely that plants have evolved to sense and respond to an endogenous, as yet hypothetical, KAI2-ligand (KL) (Waters *et al.*, 2014; Conn and Nelson, 2016). Although the chemical nature of endogenous KL remains elusive, its biological roles appear to be broader than previously appreciated, including the control of germination, de-etiolation, light-dependent vegetative growth, formation of symbiotic associations with arbuscular mycorrhizal fungi (AMF), and, identified very recently, drought adaptation (Nelson *et al.*, 2009, 2010; Sun and Ni, 2011; Gutjahr *et al.*, 2015; Li *et al.*, 2017). Despite our knowledge of the structure of KAI2 bound with KAR (Guo *et al.*, 2013), we are still far from understanding how KAI2 senses its ligands, i.e. KAR and endogenous KL. For example, it has not been resolved how the catalytic triads (Ser-His-Asp) of KAI2 participate in its ligand-binding or signaling (Waters *et al.*, 2015*b*). In addition, it remains to be determined how KL–KAI2 signaling interacts with other regulatory networks for proper developmental adjustment in higher plants.

Light is one of the most influential environment factors for plants, impacting on diverse aspects of growth and development throughout their life cycles (Smith, 2000; Chen *et al.*, 2004; Franklin and Quail, 2010; Casal, 2013). Several classes of photoreceptors have been characterized that mediate both distinct and overlapping light-dependent responses, such as photoreceptors that absorb red/far-red (phytochromes), blue-light receptors (cryptochromes, phototropins, and ZTL/FKFs), and UV-B light receptors (UVR8) (Rockwell *et al.*, 2006; Jenkins, 2014; Christie *et al.*, 2015; Galvão and Fankhauser, 2015). The downstream signaling of activated photoreceptors has been extensively studied over recent decades, providing insights into the regulatory network of light-signaling. For example, light-activated phytochromes translocalize from the cytosol into the nucleus, and turn on early transcriptional circuitry (Nagatani and Matsushita, 2002; Leivar *et al.*, 2009). Phytochrome-interacting proteins constitute diverse classes of transcriptional regulators (Bae and Choi, 2008), including a family of basic helix-loop-helix proteins, PHYTOCHROME INTERACTING FACTORs (PIF1-7) (Leivar *et al.*, 2008; Shin *et al.*, 2009), which regulate genome-wide gene expression cascades. For example, during germination of imbibed seeds, PIF1 activates direct target genes, such as *SOMNUS* (*SOM*) and the GA-signaling repressors, *RGA* and *GAI*, in the absence of light (Oh *et al.*, 2009). The elevated level of SOM mediates the induction of ABA biosynthetic-/signaling-related genes, such as *ABA1*, *NCED6/9*, *ABI3*, and *ABI5*, but down-regulates GA-biosynthetic genes, such as *GA3ox1* and *GA3ox2*. As such, light-responsive germination involves PIF1-regulated transcriptional cascades, which lead to increased GA-sensitivity/levels, but reduced ABA-sensitivity/levels. Recent genomics-based studies have also pointed to the extensive cross-talk between light-signaling and other endogenous or external stimuli (Seo *et al.*, 2009; Lau and Deng, 2010; Wang *et al.*, 2012). Indeed, several regulators of light signaling function as integrators of multiple signals, so that the output of light signaling can be co-ordinated with the ambient environment or the developmental/physiological context (de Lucas and Prat, 2014; Leivar and Monte, 2014). However, there are still many gaps in our understanding of the light-regulatory network (Galvão and Fankhauser, 2015; Wang and Wang, 2015).

As the *kai2* mutant was originally identified by its reduced light-dependent responses (Sun and Ni, 2011), it has been suggested that endogenous KL–KAI2 signaling is integrated into the light-regulatory network. Here, we present the genetic identification and characterization of a mutant in *Arabidopsis thaliana*, designated as *pleiotropic long hypocotyl2* (*ply2*), which affects multiple aspects of light responses, including photomorphogenic seedling development and germination. Identification of the molecular lesion of the *ply2* mutation revealed that it is a missense allele of *KAI2* that causes an amino acid substitution, Ala219Val. Physiological analyses together with a double-mutant analysis support the hypothesis that the pleiotropic effects of *ply2* can be ascribed to severely reduced KL–KAI2 signaling, which could be suppressed by the *smax1* mutation. Based on the results of biochemical and X-crystallographic analyses, we propose that the mutant KAI2^ply2^ protein has reduced ligand-binding activity as well as reduced structural flexibility, which thus compromise its sensitivity to KAR and endogenous KL. We discuss the structural features of KAI2^ply2^ with regard to ligand-binding and downstream signaling, together with specified roles of KL-signaling in the context of the light-regulatory network.

## Materials and methods

### Plant material

All of the Arabidopsis mutant lines used were in Col-0 background, unless otherwise stated. The *ply2* mutant was initially identified as a long-hypocotyl mutant under short-day conditions from EMS-mutagenized pools in the *gsd1-1D* mutant (Ayele *et al.*, 2014). After backcrossing four times to the Col wild-type, the *ply2* single-mutant was isolated and used for further physiological and genetic studies. The *phyA-211*, *phyB-9*, *CAB2::LUC*, and *ore9-1* lines have been described previously (Soh *et al.*, 2000; Woo *et al.*, 2001; Lee *et al.*, 2006; Kim *et al.*, 2008). The *pif1-ko* (SALK_072677), *smax1-3* (SALK_097346C), and *kai2-2* (NASC ID: N100282) seeds were obtained from the Arabidopsis Biological Resource Center (https://abrc.osu.edu/) or the Nottingham Arabidopsis Stock Centre (http://arabidopsis.info/).

### Light treatments and hypocotyl elongation assays

Hypocotyl elongation assays were performed similar to as described by Yang *et al.* (2003). Seeds were surface-sterilized and cold-treated for 2–3 d. The seeds were then sown on half-strength MS (Murashige and Skoog) medium containing 0.8% agar. The plates were placed under white light for 12 h to improve germination and then transferred to the appropriate experimental light conditions. Monochromatic light was administered using light chambers equipped with blue, red, or far-red light-emitting diodes (Good Feeling, Korea), of which the spectral peaks are 470, 660, and 730 nm, respectively. After incubation at 23 °C for an additional 4 d, the seedlings were photographed. Hypocotyl lengths were measured using the ImageJ software (http://imagej.nih.gov/ij/). Fluence rates were measured using a LI-1400A detector (International Light, USA). For hormone-sensitivity tests, an appropriate concentration of 3-methyl-2*H*-furo[2,3-*c*]pyran-2-one (karrikin_1,_ KAR_1_) or 2*H*-furo[2,3-*c*]pyran-2-one (karrikin_2,_ KAR_2_) (Toronto Research Chemicals, Canada), or *rac*-GR24 (Chiralix, Netherlands) was added before pouring the MS or aqueous medium.

### Germination assays

Germination tests were performed with seeds that had been after-ripened for at least for 2 months, essentially as described Shinomura *et al.* (1994). For phyB-dependent germination assays, seeds were surface-sterilized and sown on aqueous medium containing 0.8% agar. Seeds were irradiated with FR light (1.325 μW cm^–2^) for 15 min and then kept in darkness with or without a single pulse of R light (5.72 μW cm^–2^) for 10 min. After 5 d, the germination rate was determined based on radicle emergence. Tests were performed with three experimental replicates with at least 200 seeds for each genotype. Similar results were obtained at least twice with independent seed batches.

### Anthocyanin measurements

Arabidopsis seeds were plated on MS medium and grown under blue light (17.4 μW cm^–2^) for 3 d after irradiation with white light for 12 h. Fifty seedlings were used for each anthocyanin measurement. Total anthocyanins were determined by measuring absorption at 530 and 657 nm (A_530_ and A_657_) of the aqueous phase using a spectrophotometer, as described by Soh *et al.* (2000).

### Construction of double-mutants

Double-mutants were generated by genetic crossing. The resulting *F*_2_ population derived from selfed *F*_1_ plants was screened to select the long-hypocotyl phenotype under short-day conditions (the *ply2* phenotype) and grown to set *F*_3_ seeds. The *F*_3_ seeds were tested for the genotypes of the other mutant lines based on the mutant phenotypes or molecular markers if available, as listed in Supplementary Table S3 at *JXB* online. The selected *F*_3_ seedlings were further grown to select for homozygous lines at the next generation for double-mutant analysis. In the case of *ply2ore9-1*, increased axillary branching phenotype was used to identify the double-mutant in the *F*_3_ generation from *F*_2_ plants with a long-hypocotyl and normal branching phenotype. To confirm the genotype of *ply2*, we used a dCAPS marker, designed using the dCAPS finder (Neff *et al.*, 1998).

### RT-PCR and quantitative RT-PCR analyses

Total RNA was extracted from seeds or seedlings using a Spectrum Plant Total RNA kit (Sigma) following manufacturer’s instructions. After treatment with DNase I, 1 µg of total RNA was used for cDNA synthesis with Superscript II reverse transcriptase and an oligo(dT)_18_ primer (Invitrogen). cDNA diluted 10-fold was used for semi-quantitative RT-PCR analysis or real-time qRT-PCR analysis. Real-time qRT-PCR was conducted in an Eco™ Real-Time PCR System (Illumina), using QuantiMix SYBR Kits (PhileKorea) in a 10 µl volume. The reactions were performed in triplicate for each run. The comparative 2^–ΔΔ*C*t^ method was used to evaluate the relative quantities of each amplified product in the samples (Livak and Schmittgen, 2001). Relative expression levels were normalized according to *C*t values for *PP2A*, as described by Shin *et al.* (2015). Primer sequences are listed in Supplementary Table S3. Three biological replicates were performed for each expression analysis.

### Positional cloning of *PLY2*

To map the *PLY2* locus, we crossed *ply2* with L*er* wild-type. From the segregating *F*_2_ population thus derived, seedlings with long hypocotyls were selected and subject to further phenotypic confirmation at the following *F*_3_ generation. Extraction of genomic DNA was performed using the 125 confirmed *F*_3_ lines with the long hypocotyl phenotype under short-day conditions (10/14 h light/dark). Using PCR-based molecular markers (Shin *et al.*, 2015), initial mapping located *PLY2* at the lower arm of chromosome 4. Fine-mapping was performed using newly designed SSLP/CAPS markers, listed in Supplementary Table S3, based on Cereon Polymorphism (http://www.arabidopsis.org). The molecular lesion of *ply2* was identified by PCR amplification and sequencing analysis of genes annotated in the mapped region. For transgenic complementation of *ply2*, a full-length cDNA of wild-type *KAI2* was amplified by RT-PCR and cloned into the pENTR1A vector (Invitrogen). After verifying no sequence alteration, it was recombined into a plant overexpression vector, pH2GW7, using gateway cloning technology (Invitrogen) (Karimi *et al.*, 2002). The resulting binary vector was introduced into *Agrobacterium tumefaciens* GV3101. Transformation was performed with the *ply2* mutant according to a modified floral dip method (Clough and Bent, 1998). More than ten *T*_3_ transgenic lines with homozygous single T-DNA insertions were isolated and used for physiological analysis. Phylogenetic analysis was performed using the MEGA6 program (Tamura *et al.*, 2013), following the instructions, after retrieving sequences of KAI2 homologous proteins using the blastp program at NCBI (http://www.ncbi.nlm.nih.gov/).

### Subcellular localization assays

The full-length *KAI2* or *KAI2*^*ply2*^ ORF was amplified by PCR with primers containing appropriate restriction sites and then cloned into the pENTR1A vector (Invitrogen). After verification of no sequence errors, the entry clones were recombined into the Gateway version of pCsVMV-eGFP-N-999. For transient expression in Arabidopsis, mesophyll cell protoplasts were prepared from 3-week-old Arabidopsis Col-0 leaves and transfected with the construct using the PEG method as described previously (Kim *et al.*, 2008). Fusion protein expression was observed using Zeiss LSM 510 Meta confocal microscopy (Carl Zeiss, Germany).

### Assays of circadian rhythmic activity of *CAB2::LUC*

Plants carrying the *CAB2::LUC* reporter were grown on 1/2 B5 medium containing 1% sucrose with 12/12 h light/dark cycles under white light and transferred at ZT 0 to constant white light (20 μmol m^–2^ s^–1^). Luminescence activity was measured as described previously (Kim *et al.*, 2008). Briefly, luminescence images were recorded using a Peltier-cooled CCD camera (Versarray; Roper Scientific). Image processing and quantification were performed using the MetaVue software program (Universal Imaging). Data were imported into the Biological Rhythms Analysis Software System (BRASS v2.14) and analysed with the FFT-NLLS suite of programs. Period lengths are shown as partially variance-weighted periods (±SE), which were estimated using bioluminescence data with a time window from 24 to 96 h under free-running conditions.

### Preparation of recombinant proteins

The full-length cDNA of *KAI2* or *KAI2*^*ply2*^ was subcloned into the pET28a vector (Novagen), and the protein was expressed in *Escherichia coli* strain BL21 (DE3) (Invitrogen). Cells at mid-log phase were further grown at 20 °C for 18 h following IPTG induction and then collected by centrifugation. Cell pellets were resuspended in lysis buffer (20 mM Tris-cl pH 7.5, 150 mM NaCl, 20 mM Imidazole, 2 mM β-mercaptoethanol) and lysed by sonication. The soluble lysates were passed through a Ni-NTA sepharose column and eluted with lysis buffer supplemented with 300 mM Imidazole. The N-terminal histidine tag was removed by treatment overnight with TEV protease. The protein was further purified by size-exclusion chromatography on a Superdex 75 column, and concentrated to final concentration of 20 mg ml^–1^.

### Generation of antibody and immunoblot analysis

A polyclonal antibody was raised in rabbit against the recombinant KAI2 protein, purified from *E. coli*. For immunoblot analysis, the total proteins were extracted from approximately thirty 7-d-old seedlings grown under continuous light after treatment either with a mock solution (0.1% DMSO) or with 10 μM KAR_2_ for 2 h, essentially as described by Choi *et al.* (2014). After separation on 10% SDS-polyacrylamide gels, the proteins were transferred onto nitrocellulose membranes, and incubated with the anti-KAI2 antibody (1:1000). Antibody-bound proteins were detected by incubation with secondary antibody conjugated to horseradish peroxidase using the ECL system (Amersham Biosciences). Densitometric analysis was performed using ImageJ.

### Crystallization and determination of structure

Crystals of KAI2 and KAI2^ply2^ were grown under the same conditions by vapor diffusion at 17 °C from 1:1 mixtures of protein solution and a reservoir solution (1 M Na-citrate, 100 mM HEPES pH 7.5, 5% glycerol). Crystals were flash-frozen by immersion in liquid nitrogen, then incubated in a cryoprotectant solution (reservoir solution supplemented with 20% ethylene glycol). The observed reflections were indexed, integrated, and scaled using HKL2000 (Otwinowski and Minor, 1997). Initial crystallographic phases for KAI2 structure were determined using MOLREP (Vagin and Teplyakov, 1997) in the CCP4 suite (http://www.ccp4.ac.uk/html/molrep.html). The starting search model used a structure of RsbQ (PDB code 1WOM) (Kaneko *et al.*, 2005). *Coot* was used for visualization of electron density maps and for manual rebuilding of atomic models (Emsley and Cowtan, 2004). Refinement was performed with REFMAC in CCP4 (Vagin *et al.*, 2004).

### CPM assays

CPM assays were performed following the procedures described by Alexandrov *et al.* (2008). A stock solution of CPM dye (N-[4-(7-diethylamino-4-methyl-3-coumarinyl)phenyl]maleimide; Invitrogen) was prepared in DMSO (Sigma) at 4 mg ml^–1^ and diluted to 0.1 mg ml^–1^ in dilution buffer (20 mM HEPES pH 7.5, 150 mM NaCl) prior to use. The thermal denaturation assay was performed in a total volume of 130 μl. The KAI2 and KAI2^ply2^ proteins were diluted in the appropriate buffer to a final volume of 120 μl, adjusted to a final concentration of 51 μM. Then 10 μl of the diluted dye was added and thoroughly mixed with the protein. For the KAR-binding assay, 1000 μM (final concentration) of KAR_1_ was added. The reaction mixture was transferred within a 5 min period to a sub-micro quartz fluorometer cuvette (Starna Cells, Inc., Atascadero, CA) and heated in a controlled way with a ramp rate of 2 °C min^–1^ in a Cary Eclipse Spectrofluorometer. The excitation wavelength was set at 387 nm, while the emission wavelength was set to 463 nm. Assays were performed over a temperature range starting from 20 °C and ending at 90 °C.

### Circular dichroism spectrometry

Circular dichroism (CD) spectra for KAI2 and KAI2^ply2^ were recorded on a Jasco J-815 spectropolarimeter. Molar ellipticity was calibrated with deionized water. Temperature was controlled with a NESLAB operation temperature system connected to a NESLAB water bath RTE-100. Using 1 mg of KAI2 or KAI2^ply2^ protein in 10 mM HEPES buffer (pH 7.5), thermal denaturation profiles were obtained at 220 nm, varying the temperatures from 10 °C up to 75 °C in 2 °C increments. The data were smoothed with SigmaPlot (Jandel Scientific), then the fraction of denatured protein as a function of temperature was calculated.

### Isothermal titration calorimetry

Isothermal titration calorimetry (ITC) experiments were carried out at 25 °C in a MicroCal iTC200 calorimeter (GE Healthcare). The KAR_1_ compound dissolved in ITC buffer (20 mM HEPES pH 8.0, 50 mM sodium chloride) was injected in 2.0-μl increments into calorimetric cells containing 200 μl of KAI2 or KAI2^ply2^. The interval between injections was 150 s. Titration data were analysed using the Origin 7.0 data analysis software. Injections were integrated following manual adjustment of the baselines. Heats of dilution were determined from control experiments with the ITC buffer and subtracted prior to curve-fitting using a single set of binding site models.

### Accession numbers

The co-ordinates of KAI2 and KAI2^ply2^ and the structure factors have been deposited in the Protein Data Bank with codes 5Z9G and 5Z9H, respectively. The accession numbers of genes used in this study are listed in Supplementary Table S3.

## Results

### Isolation of a pleiotropic long-hypocotyl mutant, *ply2*

To characterize genetic components that control light-dependent seedling development, we have screened long-hypocotyl mutants in *Arabidopsis thaliana*. In this study, we present genetic identification of a mutant designated as *pleiotropic long hypocotyl2* (*ply2*). The *ply2* mutant was originally identified from EMS-mutagenized pools under short-day conditions. When we grew the seedlings under different photoperiodic conditions, the elongated hypocotyl phenotype was more pronounced under short-day conditions compared to continuous light (see Supplementary Fig. S1A). Although the photoperiod-sensitive long-hypocotyl phenotype was reminiscent of mutants with an altered circadian rhythm, such as *elf3-1* (Hazen *et al.*, 2005), the *ply2* mutation only marginally affected the circadian rhythm phenotype of *CAB::LUC* reporter activity (Supplementary Fig. S2). In line with these findings, unlike the mutants *elf3-1* or *phyB-9*, the *ply2* mutant exhibited normal flowering-time phenotypes under both long- and short-day conditions (Supplementary Fig. S1B). In addition, the *ply2* mutant exhibited low germination rates under phytochrome B (phyB)- or phytochrome A (phyA)-dependent light conditions (Shinomura *et al.*, 1994) (Supplementary Fig. S1C). We also found that the *ply2* mutant was partially defective in light-responsive expression of some, but not all, light-regulated genes, including *CAB*, *RBCS*, *STH7*, and *ELIP2* (Supplementary Fig. S1D). The pleiotropic effects suggested a critical role in light-dependent development, which led us to conduct further molecular analysis of the *PLY2* gene.

### *ply2*, a missense mutation of *KAI2* that encodes a putative KAR receptor

**Fig. 1.**
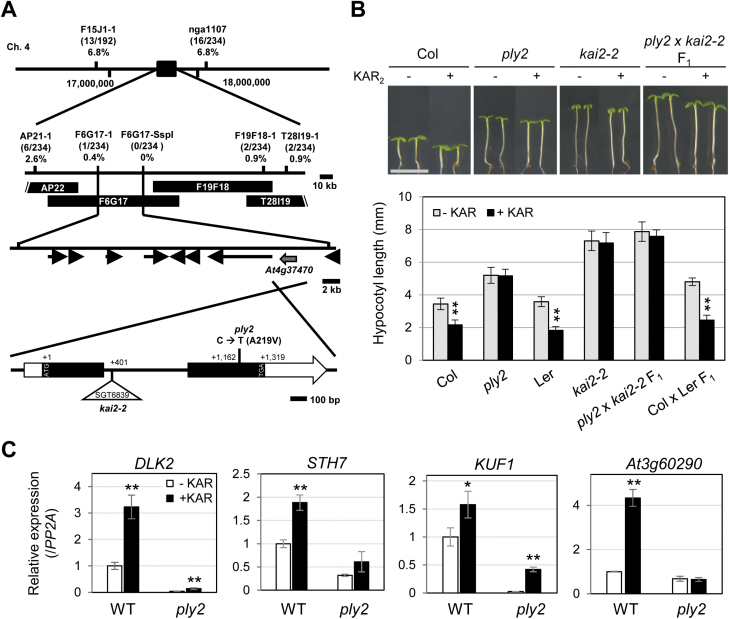
Map-based cloning of *PLY2*. (A) Schematic diagram of map-based cloning. The *PLY2* locus was initially mapped between the AP22 and T28I19-1 markers on chromosome 4. Fine-mapping narrowed the location to between the F6G17-1 and F6G17-SspI markers. The numbers in parenthesis indicate the numbers of recombinant chromatids. Candidate gene sequencing revealed that a substitution of 656th C to T occurred in the *At4g37470* gene of the *ply2* mutant, predicted to change the 219th alanine to valine (Ala219Val). The triangle indicates the transposon-insertion site in the *kai2-2* mutant. (B) Complementation test between *ply2* and *kai2-2*. The *F*_1_ plants of *ply2* and *kai2-2* were analysed for hypocotyl growth. The seedlings were sown on MS media and grown under short-day conditions (10/14 h light/dark) for 5 d with or without 10 μM KAR_2_ (KAR). The images show representative seedlings; scale bar, 5 mm. The graph shows the mean hypocotyl length (±SD) of at least 12 seedlings (*n*=12–18). Significant differences compared to the mock-treated control were determined using Student’s *t*-test; **P<0.01. (C) KAR-responsive gene expression in the *ply2* mutant. Seedlings were grown under continuous light for 5 d. After treatment with either 10 μM KAR_2_ (+KAR) or 0.01% DMSO as control (–KAR) for 3 h, total RNAs were extracted and subjected to real-time RT-PCR analysis. The data are mean (±SD) values of relative expression level from experimental replicates (*n*=3), normalized to the level of *PP2A* expression in mock-treated wild-type (Col). Significant differences from the mock-treated control were determined using Student’s *t*-test; **P<0.01, *P<0.05.

Genetic analysis indicated that the long-hypocotyl phenotype of *ply2* under short-day conditions was inherited recessively. Subsequent *F*_2_ analysis showed a typical Mendelian segregation ratio of 3:1 (wild-type to mutant, 168:56), indicative of the monogenic nature of the *ply2* mutation. To further understand the molecular nature of the *PLY2* gene, we performed map-based cloning. Fine-mapping combined with candidate gene sequencing revealed that the *ply2* mutant carried a C to T substitution mutation in the coding region of the *At4g37470* gene, predicted to change Ala219 to Val in an α/β hydrolase fold protein (Fig. 1A). Next, we performed transgenic complementation by overexpressing the wild-type full-length cDNA of *At4g37470* in the *ply2* mutant. Multiple independent transgenic lines restored the long-hypocotyl phenotype as well as the defective light-induced germination phenotypes of the mutant (see Supplementary Fig. S3). These results confirmed that the missense mutation of *At4g37470* was the responsible molecular lesion of the *ply2* mutant. Two independent studies have identified the function of the same gene, *At4g37470*, designating it as *HYPOSENSITIVE TO LIGHT* (*HTL*) or *KARRIKIN-INSENSITIVE2* (*KAI2*), which hereafter we refer to as *KAI2* (Sun and Ni, 2011; Waters *et al.*, 2012). To further confirm that the *ply2* mutant phenotype was due to mutation of *KAI2*, we performed a complementation test between *ply2* and *kai2-2*, a knock-out allele of *KAI2*. The *F*_1_ seedlings derived from crossing *ply2* and *kai2-2* exhibited a KAR-insensitive, long-hypocotyl phenotype, as did the parental single-mutants, indicating that *ply2* is allelic to *kai2-2* (Fig. 1B). The KAR-insensitivity of the *ply2* mutant was also assessed with regards to the expression of the KAR-responsive genes *STH7*, *DLK2*, *KUF1*, and *At3g60290* (Nelson *et al.*, 2010; Stanga *et al.*, 2013). As shown in Fig. 1C, the basal expression of these genes was down-regulated in the *ply2* mutant compared with the wild-type. Furthermore, the KAR-inducibility of the genes was severely, if not completely, compromised in the *ply2* mutant, suggesting a critical defect of *KAI2*^*ply2*^ in the KAR-responsive gene expression. These results suggested that the missense mutation Ala219Val of the *KAI2* gene caused defects in KAR-sensitivity as well as in multiple aspects of light-responses in Arabidopsis.

### Expression profiling of germination-related genes

**Fig. 2.**
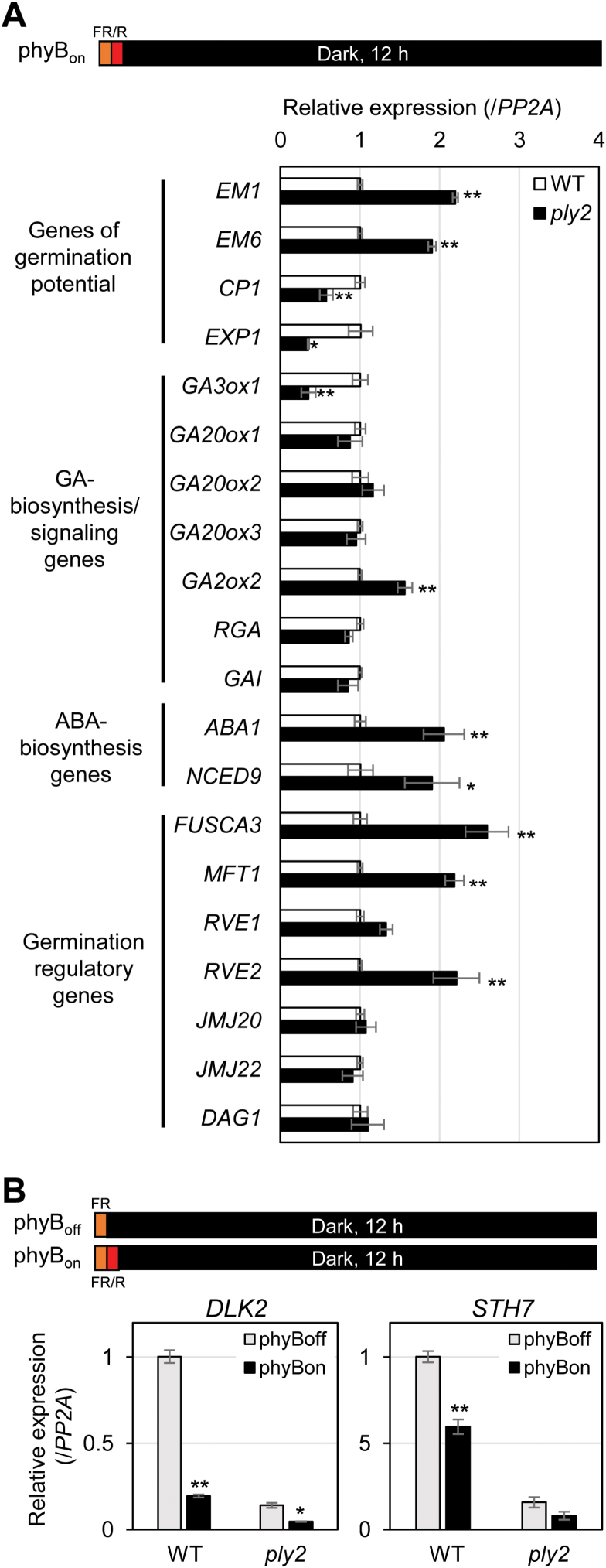
Gene expression profiling during phytochrome B (phyB)-dependent germination. The experimental scheme is depicted at the top. For analysis of phyB-dependent gene expression, seeds were irradiated with far-red light for 15 min without (phyB_off_) or with subsequent red light for 10 min (phyB_on_). After incubation in darkness for 12 h, total RNA was extracted and subject to real-time RT-PCR analysis. *PP2A* was used as the control. (A) Expression of germination-related genes under phyB_on_ conditions. The relative expression of each gene in the *ply2* mutant was normalized to that in the wild-type (WT), which was set to 1. Data are means (±SD) of experimental replicates (*n*=3). Significant differences from the expression level of the wild-type were determined using Student’s *t*-test; **P<0.01, *P<0.05. Similar trends were found with two independent seed batches and are shown in Supplementary Fig. S4. (B) Expression of the KAR-target genes *DLK2* and *STH7* under both phyB_off_ and phyB_on_ conditions. The relative expression level of each gene was normalized to that of the wild-type under phyB_off_ conditions, which was set to 1. Data are means (±s.e.m.) from experimental replicates (*n*=6) with two independent seed batches. Significant differences from the expression level under phyB_off_ conditions were determined using Student’s *t*-test; **P<0.01, *P<0.05. (This figure is available in colour at *JXB* online.)

Several independent studies have reported the involvement of KL–KAI2 signaling in seed germination and light-dependent seedling development (Shen *et al.*, 2007; Nelson *et al.*, 2011; Sun and Ni, 2011; Waters *et al.*, 2012). Shen *et al.* (2012) also showed that MAX2 controls phyB-dependent gene expression during germination in a PIF1-independent way, presumably through KL-signaling. To examine the effects of *ply2* on light-responsive germination at the molecular level, we performed expression profiling under phyB_on_ light conditions (treatment with FR light pulse/R light pulse followed by darkness) for genes with light-regulated expression (Fig. 2A and Supplementary Fig. S4). In accordance with the reduced germination rate, genes correlating with dormancy level or germination potential such as *EM1*, *EM6*, *CP1*, and *EXP1* were significantly up- or down-regulated in the *ply2* mutant. As shown in Fig. 2A, the expression of several genes including *GA3ox1*, *GA2ox2*, *ABA1*, *NCED9*, *FUSCA3*, *MFT1*, and *RVE2* were significantly affected in the *ply2* mutant compared to wild-type, being down- or up-regulated by at least 1.5-fold. In contrast, the *ply2* mutation did not significantly affect the transcript level of other germination regulatory genes, including *RGA*, *GAI*, *GA20ox1*, *GA20ox2*, *GA20ox3*, *JMJ20*, *JMJ22*, *DAG1*, and *RVE1*. Together, these results showed that the reduced germination rate of *ply2* under phyB_on_ light conditions was accompanied by transcriptional changes of a subset of light-regulated genes, which included several ABA/GA-biosynthetic genes as well as germination regulatory genes. Notably, the altered expression profiles of the *ply2* mutant largely overlapped with those of *max2*, as reported by Shen *et al.* (2012). In line with the hypothesis that KAI2–MAX2 mediated KL-signaling acts in a PIF1-independent pathway, we also found that *ply2* could suppress the independent germination phenotype of the *pif1* mutant (see Supplementary Fig. S5). Interestingly, when we investigated the effects of light on the expression of the primary KAR-target genes *DLK2* and *STH7*, we found that they were down-regulated by red-light treatment (phyB_on_ conditions). In the *ply2* mutant, the reduced expression of *DLK2* was further down-regulated by red-light treatment (Fig. 2B). The results implied that light and KAI2-signaling act largely independently with differential modes of interaction, functioning additively or oppositely, depending on the target genes.

### Double-mutant analysis of *ply2ore9-1* and *ply2smax1-3*

**Fig. 3.**
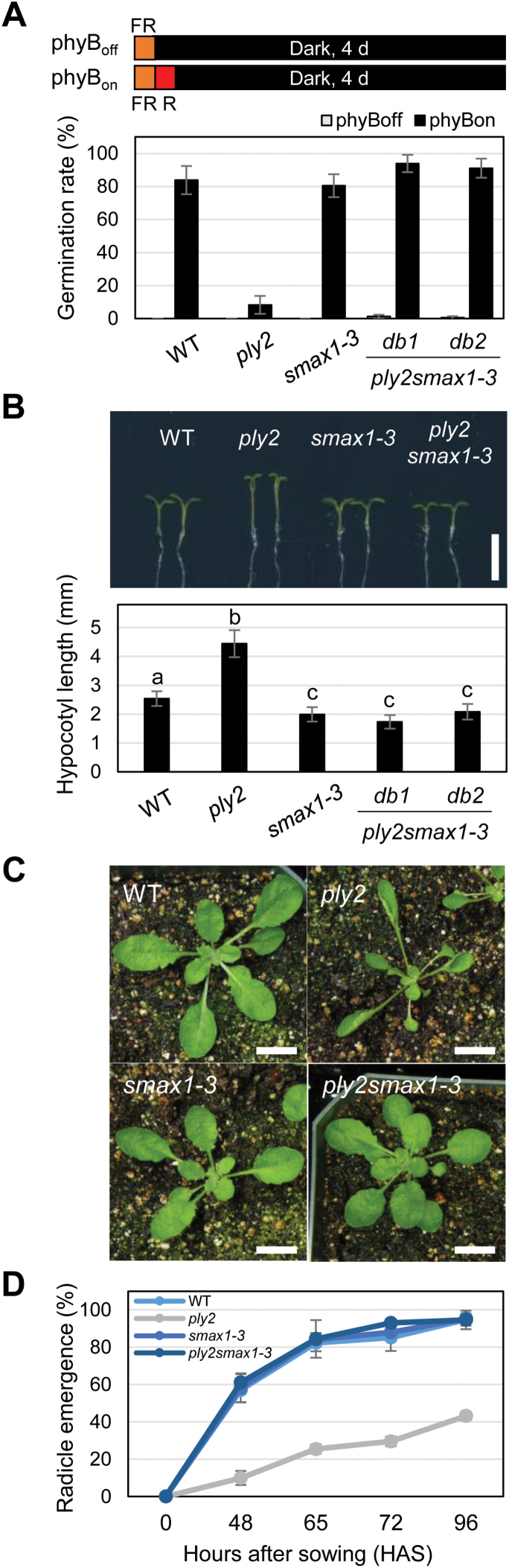
Double-mutant analysis of *ply2* and *smax1*. (A) Phytochrome B (phyB)-dependent germination of the wild-type (WT, Col), *ply2*, *smax1-3*, and *ply2smax1-3* double-mutant. After treatment with far-red light for 10 min, the seeds were then either irradiated (phyB_on_) or not (phyB_off_) with red light for 10 min, and then kept in darkness for 4 d. Mean values (±SD) of germination rates were determined from three experimental replicates with at least 50 seeds per plate. (B) Hypocotyl elongation assay. The images show representative 6-d-old seedlings of each genotype grown on half-strength MS-SUC (0.5%) media under short-day conditions (10/14 h light/dark); scale bar, 5 mm. The graph shows the mean (±SD) hypocotyl length of the seedlings (*n*=15–18). Different letters indicate significant differences at *P*<0.01, analysed by one-way ANOVA with a *post hoc* Tukey HSD test. (C) Leaf morphology of 30-d-old plants. The plants were grown under short-day conditions (10/14 h light/dark); scale bars, 10 mm. (D) Primary dormancy assay. Freshly harvested seeds of each genotype were sown on aqueous media and incubated under continuous light conditions. Mean values (±SD) of germination rate were determined at different times after sowing for three experimental replicates with at least 50 seeds per plate.

The *SUPPRESSOR OF MAX2 1* (*SMAX1*) was originally identified as a genetic suppressor of the *max2-1* mutant (Stanga *et al.*, 2013) that rescues the KAR-dependent phenotypes of the mutant, while not affecting SL-dependent defects such as increased branching. Based on the working hypothesis that KAI2 acts together with MAX2-SMAX1 in the KL-signaling pathway (Morffy *et al.*, 2016), we hypothesized that the *smax1-3* mutant would restore all of the developmental defects of the *ply2* mutant. To assess this hypothesis, we generated the double-mutant *ply2smax1-3* by genetic crossing. The reduced germination phenotype of *ply2* under phyB_on_ light conditions was restored in the double-mutant (Fig. 3A). In addition, other phenotypic defects of *ply2*, including the long hypocotyl, elongated petiole, and deep seed dormancy, were also suppressed by the *smax1-3* mutation (Fig. 3B–D). These results were in good agreement with the hypothesis that SMAX1 acts as a negative regulator downstream of KL–KAI2-signaling.

To substantiate the hypothesis that the pleiotropic defects of *ply2* are due to impaired KL–KAI2-signaling, we performed a double-mutant analysis of *ply2* and the *ore9-1* mutant, a null allele of *MAX2* (Woo *et al.*, 2001). The results showed that the *ply2ore9-1* double-mutant exhibited essentially the same responses compared to the parental single-mutants, *ply2* and *ore9-1*, without any noticeable additivity or synergism with regards to KAR-responsive hypocotyl elongation and light-responsive anthocyanin accumulation (Supplementary Fig. S6).

### Functional aspects of the *KAI2*^*ply2*^ mutation

Given the phenotypic severity of the *ply2* mutant, which was as severe as a null-mutant of *KAI2* or *MAX2* (Fig. 1, Supplementary Fig. S6), it was intriguing how a single amino acid substitution, Ala219Val, could compromise the function of KAI2. It is notable that the mutated residue in *ply2*, Ala219, is one of the highly conserved amino acids specifically among KAI2-clade homologous proteins (see Supplementary Fig. S7). Given that it has been proposed that the AtD14 and KAI2 clade proteins have evolved to sense and mediate distinct downstream signaling of different ligands, SL and KL, respectively (Conn *et al.*, 2015; Toh *et al.*, 2015; Waters *et al.*, 2015*b*; Xu *et al.*, 2016; Yao *et al.*, 2017), it seemed plausible that the mutated residue might be functionally critical for ligand-specific binding/signaling. Therefore, we investigated the molecular and biochemical features of KAI2^ply2^ to determine how KAI2^A219V^ affects its receptor function.

**Fig. 4.**
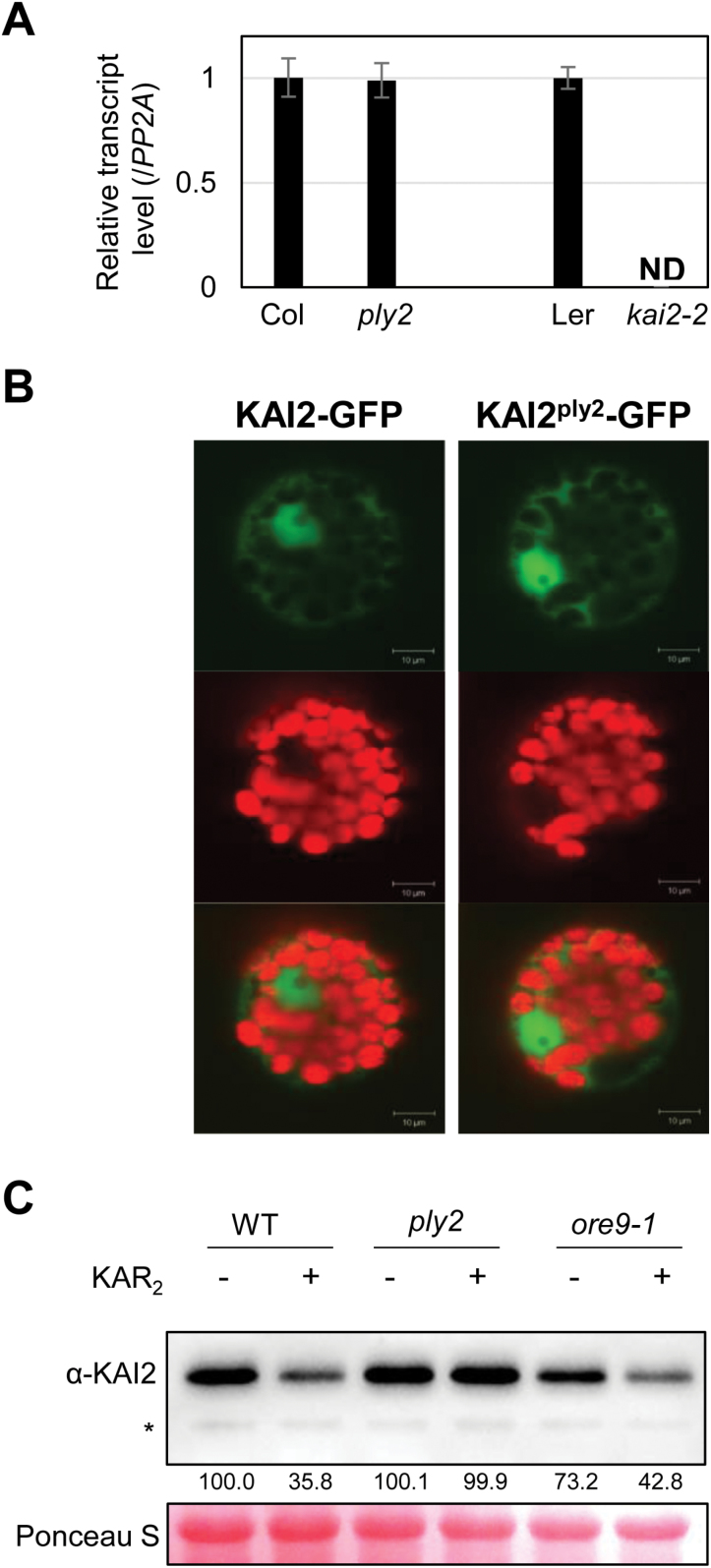
Functional assay of the *KAI2*^*ply2*^ mutation. (A) Expression analysis of the *KAI2* transcript. Total RNA from 5-d-old light-grown seedlings was used for qRT-PCR analysis. *PP2A* was used as a loading control. The relative expression of the *KAI2* transcript in the *ply2* or *kai2-2* mutant was compared to that in the wild-type, which was set to 1. Data are means (±SD) of experimental replicates (*n*=3). ND, not detected. (B) Subcellular localization of KAI2. The wild-type *KAI2-GFP* or *KAI2*^*ply2*^*-GFP* constructs were transfected to Arabidopsis mesophyll protoplasts. Representative images taken by confocal microscopy are shown: GFP signal (upper), chloroplast auto-fluorescence (middle), merged image (lower). (C) Immunoblot analysis of the KAI2 protein. Total proteins were extracted from seedlings grown under continuous light for 7 d after treatment with a mock solution (–) or 10 μM KAR_2_ (+) for 2 h. The KAI2 protein (29.8 kDa) was detected using an α-KAI2 antibody. The transferred proteins were stained with Ponceau S solution as a loading control. Numbers below the bands indicate the relative densitometry values of the KAI2 protein band, normalized to those of the loading control. The asterisk indicates a non-specific protein.

Expression analysis showed no significant differences in the *KAI2* transcript level between the wild-type and *ply2* mutant, precluding any effect of the mutation on the transcriptional expression of *KAI2* (Fig. 4A). Next, we examined whether the *ply2* mutation altered the subcellular localization of KAI2. After transient expression of *KAI2-GFP* or *KAI2*^*ply2*^*-GFP* constructs in protoplasts, we performed confocal microscopy analysis. As previously reported by Sun and Ni (2011), both KAI2 and KAI2^ply2^ were shown to be localized in the cytosol as well as in the nucleus (Fig. 4B), indicating that the *ply2* mutation did not alter the subcellular localization of KAI2. When we performed immunoblot analysis, contrary to expectation, the amount of KAI2^ply2^ protein was not altered in the *ply2* mutant compared to the wild-type (Fig. 4C). Taking into consideration recent results indicating that KAR triggers degradation of KAI2 (Waters *et al.*, 2015*a*), we also checked whether the *ply2* mutant was defective in this regard, and found it to be the case. The results suggested that the increased level of KAI2^ply2^ in the *ply2* mutant might have resulted from defects in signaling for KAI2 turnover, which would constitute a negative feedback regulation of KAI2-signaling.

**Fig. 5.**
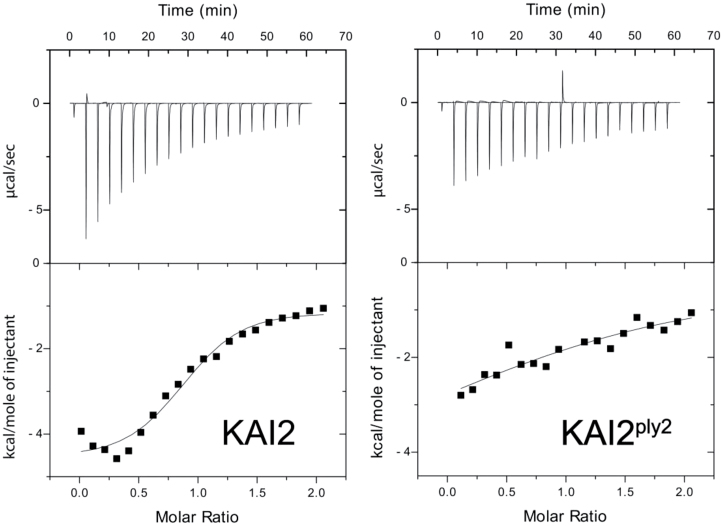
Reduced ligand binding activity of KAI2^ply2^. Isothermal titration calorimetry (ITC) experiments were performed by titrating KAR_1_ into recombinant KAI2 or KAI2^ply2^ protein. The upper panels show raw ITC data. The lower panels present data for the enthalpy change derived from the upper panels. Corresponding thermodynamic properties of KAI2 and KAI2^ply2^ are shown in Supplementary Table S1.

Being a KAR-receptor, the KAI2 protein has been shown to bind with KAR by various biochemical assays (Guo *et al.*, 2013; Kagiyama *et al.*, 2013; Toh *et al.*, 2014). To assess the possibility that KAI2^ply2^ was defective in ligand-binding activity, an isothermal titration calorimetry (ITC) assay was used to determine the binding properties of KAI2 and KAI2^ply2^ towards KAR_1_ (3-methyl-2*H*-furo[2,3-*c*]pyran-2-one). As shown in Fig. 5, KAI2 and KAI2^ply2^ exhibited different thermodynamic properties upon application of KAR_1_. The results showed that the dissociation constant (*K*_d_) of KAI2^ply2^ towards KAR_1_ was dramatically increased to 2857 μM, about 20-fold higher than that of KAI2 (147 μM) (see Supplementary Table S1), indicating that the KAI2^ply2^ mutation impaired its binding activity for KAR. Under the same experimental conditions, the KAI2^S95A^ mutant protein displayed very low (indeed hardly detected) binding activity toward KAR_1_ (results not shown), supporting the idea that the Ser95 residue, one of the catalytic triad of KAI2, is critical for the biological function and ligand-binding activity (Waters *et al.*, 2015*b*). The results suggested that the mutant protein KAI2^ply2^ had greatly reduced ligand-binding activity. We also assessed the ligand-binding activity by a N-[4-(7-diethylamino-4-methyl-3-coumarinyl)phenyl]maleimide (CPM) assay. Upon addition of KAR_1_, the wild-type KAI2 protein exhibited a shifted thermal function of fluorescence, indicating that KAI2 undergoes changes in conformational flexibility caused by KAR_1_ (Supplementary Fig. S8). In contrast, KAI2^ply2^ showed only a marginal change upon application of KAR_1_, implying that KAI2^ply2^ was defective in ligand-induced conformational changes. It was also notable that the KAI2 and KAI2^ply2^ proteins exhibited different values for melting temperature (*T*_m_) in the absence of KAR_1_; the *T*_m_ values of KAI2 and KAI2^ply2^ were calculated to be 36.39 °C and 45.60 °C, respectively, indicating that thermal stability of KAI2^ply2^ was increased (see below).

### Structural comparison of KAI2 and KAI2^ply2^

**Fig. 6.**
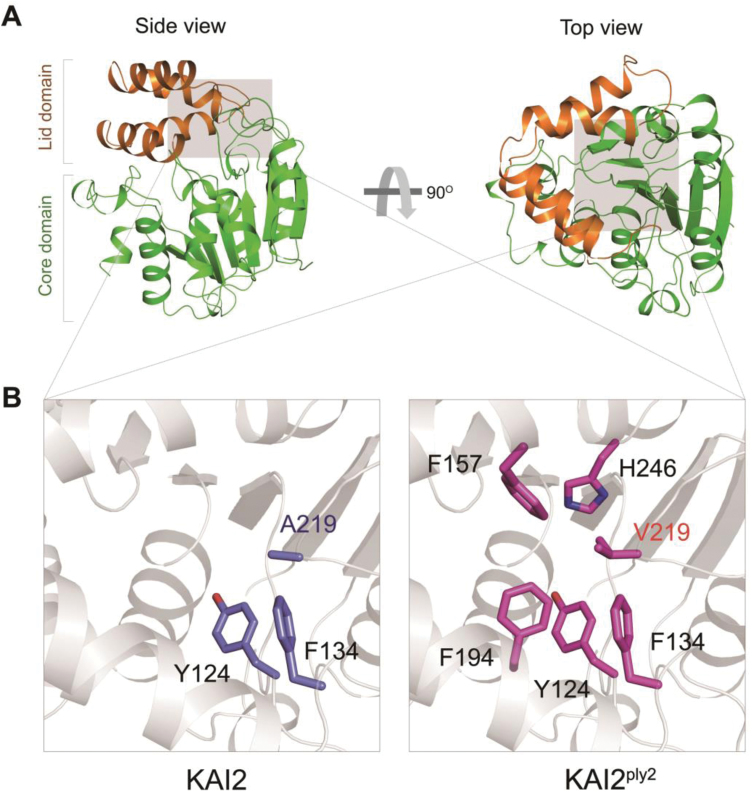
Three-dimensional X-ray crystal structure of KAI2 and KAI2^ply2^. (A) Left, a side view of KAI2. The ribbon representation of the overall structure of KAI2 is shown. Brown and green represent the lid and core domains, respectively. Right, a top view of KAI2: a 90° rotation of KAI2 relative to the side view is shown. (B) Magnified views of the areas in (A) shaded grey, showing residues forming hydrophobic interactions with A219 of KAI2 (left, indicated in blue) and V219 of KAI2^ply2^ (right, indicated in magenta).

**Fig. 7.**
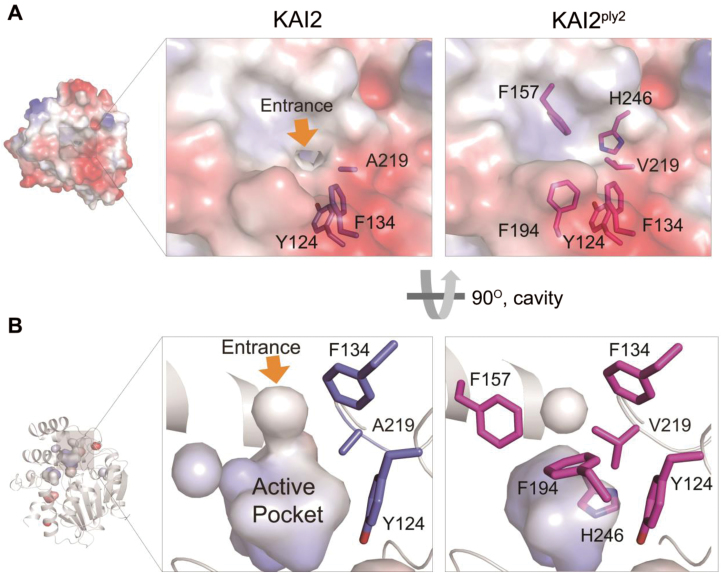
Structural comparison of KAI2 and KAI2^ply2^. (A) Surface views of KAI2 and KAI2^ply2^. KAI2 exhibits a small opening at the top, forming an entrance hole into the ligand-binding pocket (yellow arrow). Note that the entrance is closed in the KAI2^ply2^ protein. (B) Close-up view of the cavity on the active, putative ligand-binding pocket. Note that KAI2 shows an open-gate conformation with continuous space, whereas KAI2^ply2^ shows a closed gate with discontinuous space.

**Fig. 8.**
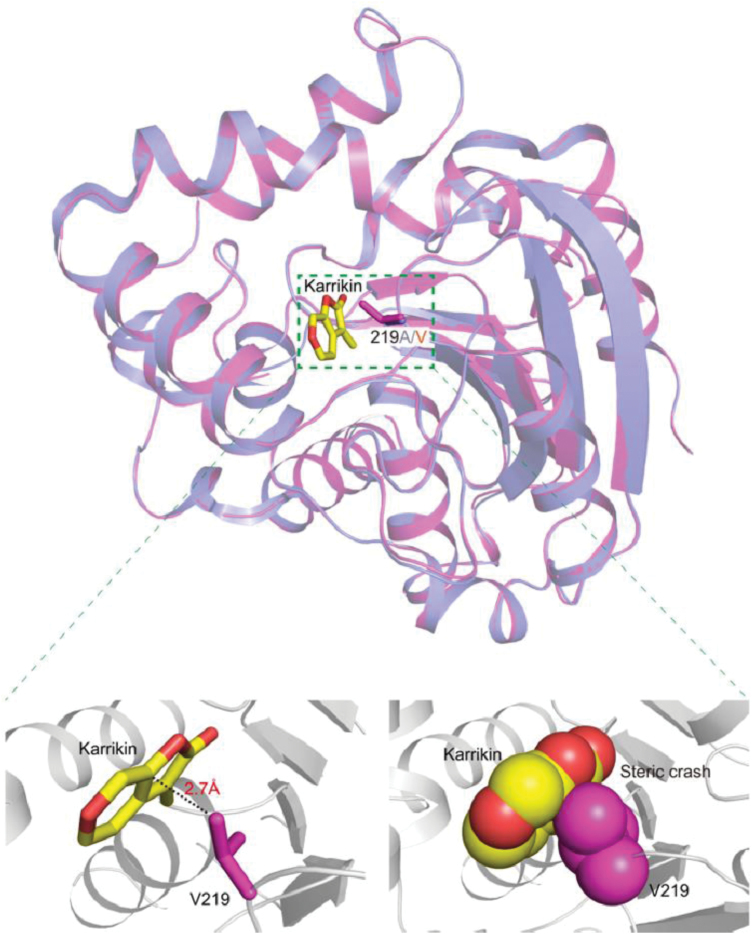
Structural comparison between KAI2^ply2^ and the KAI2–KAR_1_ complex (PDB:4JYM). Superimposition of KAI2^ply2^ (magenta) and KAI2–KAR_1_ (light blue; karrikin, yellow). The r.m.s.d. score between KAI2^ply2^ and KAI2-KAR_1_ is 0.2. The close-up views (below) shows a stick (left) and a sphere (right) model of KAR_1_ and the Val219 residue.

To gain further insight into the molecular lesion of the KAI2^ply2^ mutant protein, we performed X-ray crystallography to assess any structural alterations. Recombinant proteins of KAI2 and KAI2^ply2^, purified from *E. coli*, were crystallized. The crystal structures were solved and refined at 1.9 Å by molecular replacement (MR) using a search model based on the bacterial signaling protein RsbQ (PDB ID code 1WOM) (Kaneko *et al.*, 2005) (Supplementary Table S2). As reported previously (Bythell-Douglas *et al.*, 2013; Guo *et al.*, 2013; Kagiyama *et al.*, 2013; Zhao *et al.*, 2013; Xu *et al.*, 2016), the structure of KAI2 displayed a canonical compact α/β-hydrolase domain that contained an α/β ‘core’ domain and a ‘lid’ domain (Fig. 6A) (Nardini and Dijkstra, 1999). Comparison of KAI2 and KAI2^ply2^ showed that there was little structural difference (r.m.s.d., 0.2 Å) in overall structure. However, in detail, we found that the residue A219 of KAI2 was bound to aromatic residues as Y124 and F134 of the lid domain by hydrophobic interactions, while the residue V219 of KAI2^ply2^ was bound to extended aromatic residues as Y124, F134, F157, F194, and H246 of the lid domain (Fig. 6B). Thus, the mutation Ala219Val caused stronger hydrophobic interactions with more residues. Moreover, the electrostatic potential surface model and cavity view showed another structural alteration of KAI2^ply2^. The entrance of KAI2 presents an opened pocket and hydrophobic charge for the ligand, whereas the entrance of KAI2^ply2^ forms a closed pocket due to C-gamma 1 and C-gamma 2 of V219, which is a bulkier residue than alanine (Fig. 7). Thus, the narrowed entrance gate might contribute to the reduced ligand-binding ability of KAI2^ply2^. Furthermore, when we performed superimposition with the KAR_1_-bound KAI2 model previously reported by Guo *et al.* (2013), the V219 was predicted to cause a steric clash with the bound KAR (Fig. 8). Thus, collectively, these structural alterations may underlie the reduced binding affinity of KAI2^ply2^ to KAR (Fig. 5). While it is still open to question how KL binds to KAI2, the A219 residue may take part in ligand-specific binding of KAI2.

**Fig. 9.**
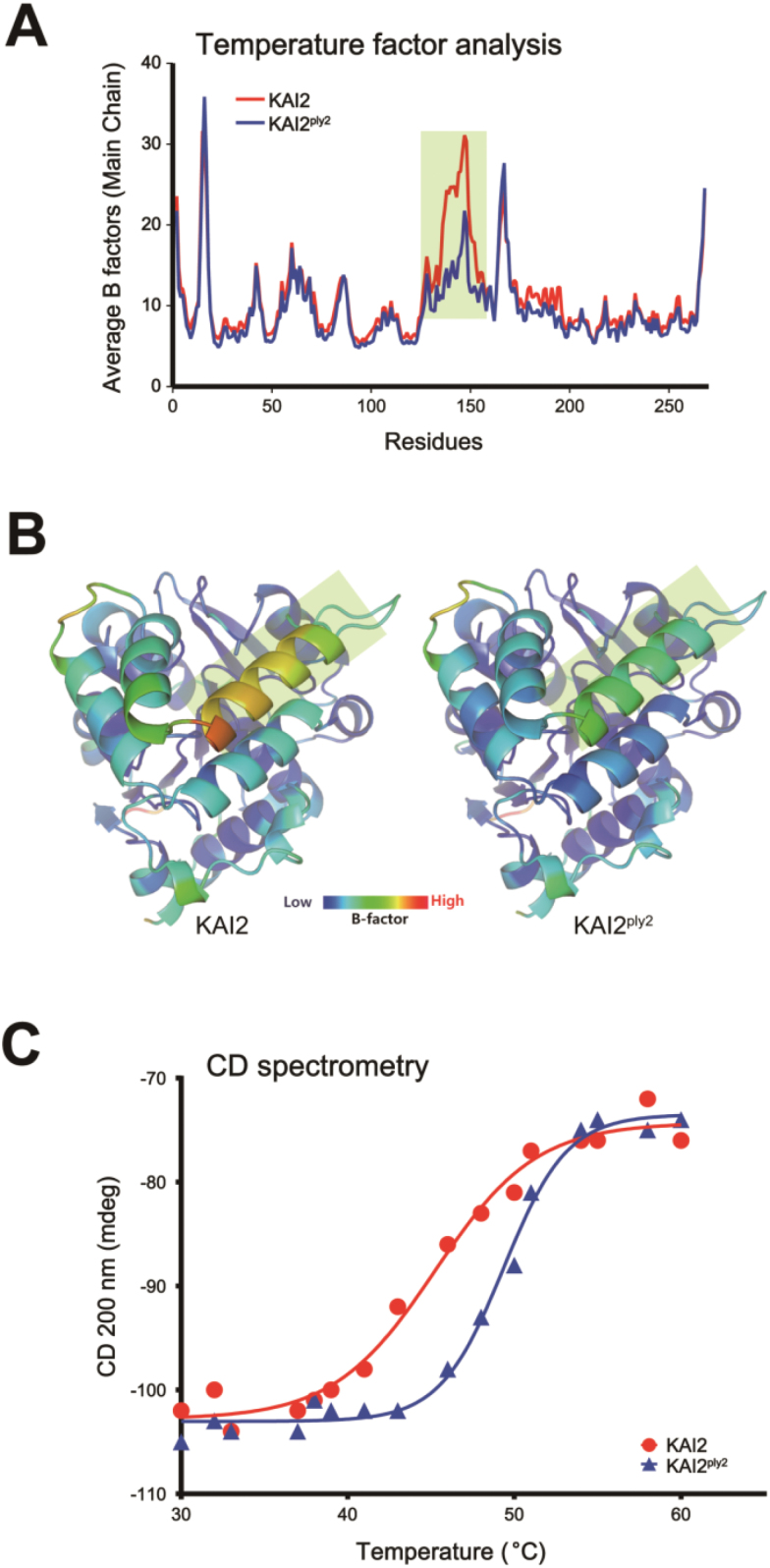
Comparison of thermal stability between KAI2 and KAI2^ply2^. (A) B-factor analysis of protein crystals for KAI2 and KAI2^ply2^. The residues ranging from 120–150 (highlighted) of KAI2 showed higher B factor values compared to those of KAI2^ply2^. (B) Ribbon diagram of B-factors of KAI2 and KAI2^ply2^. Note that the residues showing clear differences between KAI2 and KAI2 ^ply2^ in (A) correspond to the helix of the cap domains (shaded areas). (C) Circular dichroism (CD) spectrometry assay. The melting temperature (*T*_m_) of KAI2 is 45 °C and for KAI2^ply2^ it is 50 °C.

To investigate any structural impacts of the predicted increased hydrophobic interaction in KAI2^ply2^ (Fig. 6B), we performed B-factor analysis with electron density maps from protein crystals. The results showed that the amino acid residues of KAI2 ranging from 125^–^150 exhibited a higher B-factor than those of KAI2^ply2^, implying that this region, corresponding to the helical ‘lid’ domain, might be more rigid with lower motional amplitude in the KAI2^ply2^ protein (Fig. 9A, B). To assess the predicted biochemical features of KAI2^ply2^, we examined the thermal stability of KAI2 and KAI2^ply2^ by circular dichroism (CD) spectroscopy. We found that the *T*_m_ of the KAI2^ply2^ protein was increased compared to that of the wild-type KAI2 under CD assay conditions; the *T*_m_ values of KAI2 and KAI2^ply2^ were calculated to be 45.23 °C and 49.36 °C, respectively (Fig. 9C). The results suggested that KAI2^ply2^ was more thermally stable than the wild-type KAI2 protein, presumably due to increased hydrophobic interactions and reduced flexibility. Taken together, these results suggested that the Ala219Val substitution mutation of KAI2 not only impaired ligand-binding but also reduced the conformational flexibility of the lid domain, both of which would be critical for ligand perception and downstream signaling.

## Discussion

A hypothetical molecule, termed KL, for KAI2-ligand, has recently been recognized as a potentially new plant hormone that is sensed by a receptor, KAI2 (Flematti *et al.*, 2013; Conn and Nelson, 2016). Despite its unknown chemical nature and its biosynthetic pathway, a series of molecular genetic analyses of the *kai2* mutant have uncovered the biological functions of KL, including promotion of seed germination, reduced seedling de-etiolation under light conditions, drought adaptations, and formation of AMF associations (Waters *et al.*, 2014; De Cuyper *et al.*, 2017). However, the detailed mode of action of KAI2 in the ligand-binding and signaling remains to be identified.

In this study, we showed that the *ply2* mutation, a missense allele with Ala219Val substitution, caused loss of function of KAI2 (Fig. 1, Supplementary Figs S1, S3). The pleiotropic developmental defects of *ply2* were reminiscent of phenotypes of mutants defective in KL-signaling, *kai2* and *max2*, as reported previously (Shen *et al.*, 2007; Sun and Ni, 2011; Waters *et al.*, 2012; Toh *et al.*, 2014) (Figs 1, 2, Supplementary Figs S1, S5). In addition, the genetic epistasis between *ply2* and *smax1-3* suggested that the pleiotropic defect of *ply2* is due to impaired KL-signaling (Fig. 3), providing genetic evidence that that SMAX1 acts in a pathway downstream of KAI2 as a negative regulator of KL-signaling (Stanga *et al.*, 2013).

It was notable that the phenotypic severity of the *ply2* mutation was comparable to *kai2-2* and *ore9-1*, null-mutants of *KAI2* and *MAX2*, respectively (Fig. 1, Supplementary Fig. S5), suggesting that *KAI2*^*ply2*^ is a strong, if not null, loss-of-function allele. Intriguingly, the Ala219 of KAI2 is highly conserved in the KAI2-clade proteins among KAI2 homologs (see Supplementary Fig. S7) and has been annotated as one of the putative substrate-determining residues among KAI2/D14 family proteins (Chevalier *et al.*, 2014). Our results from biochemical and structural analyses provided several clues as to how the single amino acid substitution (Ala219Val) impairs the receptor function of KAI2. ITC experiments with recombinant KAI2 and KAI2^ply2^ proteins indicated that the KAR-binding activity of KAI2 was greatly (~20-fold) reduced by the *ply2* mutation (Fig. 5, Supplementary Table S1). The reduced KAR-binding ability of KAI2^ply2^ was also verified by CPM assays (Supplementary Fig. S8). In addition, X-ray crystallography studies including B-factor analyses showed that KAI2^ply2^ forms a narrower gate for the entrance into the ligand-binding pocket, and forms a more rigid helical lid domain, presumably due to enhanced hydrophobic interactions, which do not affect the overall structural integrity (Figs 6, 7, 9). Thus, the Ala219 of KAI2 appears to be important in forming space for entry of the ligand as well as maintaining flexibility of the lid domain. By analogy with the current working model of strigolactone and its receptor, D14 (Yao *et al.*, 2016), it is conceivable that the lid domain of KAI2 may take part in a ligand-dependent protein–protein interaction. In this scenario, the more rigid lid domain of KAI2^ply2^ would hamper the conformational changes upon ligand-binding, resulting in reduced KAI2 signaling. Furthermore, our homology modelling of KAI2^ply2^ with the KAR_1_-bound KAI2 model proposed by Guo *et al.* (2013) suggested that the substituted Val219 might impair ligand binding through steric hindrance (Fig. 8). However, this should be considered with caution, as it has not been unequivocally demonstrated that the KAR_1_-bound KAI2 model represents the active state of KAI2. Overall, these results indicate that the single missense mutation Ala219Val altered multiple biochemical features of KAI2^ply2^, accounting for its severe, if not complete, insensitivity to KAR or endogenous KL.

To date, only a few missense mutations of KAI2 have been characterized, including G133E and S95A (Waters *et al.*, 2015*b*). While G133 was shown to be important for protein integrity, S95, together with amino acids forming the catalytic triad of the α/β hydrolase fold, has been suggested to be critical for ligand binding. However, it remains unknown whether S95 catalyses hydrolysis of the pro-ligand, as D14 does with SL (Yao *et al.*, 2016) or if it participates in binding directly with the ligand via bridging molecule, as ShKAI2iB does (Xu *et al.*, 2016). Our identification of A219 as a critical residue for ligand binding and possibly signaling should help to determine the binding mode of KL–KAR–KAI2. Since the endogenous KL has not yet been identified, it is still speculative how KAI2 senses its ligand in order to transmit downstream signaling. Characterization of additional informative missense mutations of KAI2 by structure-guided design, mutant screening, or targeting-induced local lesions in genomes (TILLING) would help to further identify the molecular consequences during ligand perception and signal transmission of KAI2, as elegantly demonstrated by recent analysis of the *d14-5* allele, AtD14^G158E^, a missense mutant of a SL receptor (Yao *et al.*, 2016).

Taking together with recent studies showing that KAI2-signaling is also required for AMF formation and drought tolerance (Gutjahr *et al.*, 2015; Li *et al.*, 2017), it is tempting to speculate that endogenous KL levels and subsequent KAI2-signaling may represent a hormonal/metabolic signal that sensitizes or desensitizes plants to various environmental fluctuations. For example, as shown in Fig. 2 and by Shen *et al.* (2012), the loss of KL–KAI2 signaling may set a higher threshold of light for phytochrome-dependent seed germination by up-regulation of germination-inhibitory genes such as *FUSCA3*, *RVE2*, *GA2ox2*, and *ABA1* as well as by down-regulation of germination-promoting genes such as *GA3ox1*. As such, KAI2-signaling may intersect with other endogenous/environmental stimuli, controlling a subset of downstream target genes. More sophisticated experimental designs together with identification of genome-wide KL-target genes under specific physiological conditions will shed light on the additional regulatory functions of KL–KAI2-signaling in higher plants.

## Supplementary data

Supplementary data are available at *JXB* online.

Fig. S1. Identification of the *ply2* mutant with a long-hypocotyl phenotype.

Fig. S2. The circadian rhythm phenotype of the *ply2* mutant.

Fig. S3. Transgenic complementation of the *ply2* mutant.

Fig. S4. Additional biological replications of gene expression profiling under phyB-dependent germination conditions.

Fig. S5. Double-mutant analysis using *ply2* and *pif1* mutants.

Fig. S6. Double-mutant analysis using *ply2* and *ore9-1* mutants.

Fig. S7. Multiple sequence alignments of the KAI2, AtD14, and DLK2 homologs.

Fig. S8. CPM assays of KAI2 and KAI2^ply2^.

Table S1. Thermodynamic properties determined by ITC.

Table S2. Statistics for X-ray data collection and refinement.

Table S3. Primers used in this study.

Supplementary Figures and Tables

## Abbreviations:

CPM: N-[4-(7-diethylamino-4-methyl-3-coumarinyl)phenyl]maleimide
FR: far-red
ITC: isothermal titration calorimetry
KAI2: KARRIKIN INSENSITIVE2
KAR: karrikin
KL: KAI2-ligand
MAX2: MORE AXILLARY GROWTH2
MS medium: Murashige–Skoog medium
phyB: phytochrome B
PIF1: PHYTOCHROME INTERACTING FACTOR1
PLY2: PLEIOTRIPIC LONG HYPOCOTYL2
R: red
SL: strigolactone
SMAX1: SUPPRESSOR OF MAX2 1
