# Supplementary material for: A missense allele of KARRIKIN-INSENSITIVE2 impairs ligand-binding and downstream signaling in Arabidopsis thaliana
Source: J Exp Bot. 2018 May 2;69(15):3609–23. doi: 10.1093/jxb/ery164 (PMC6022639; doi:10.1093/jxb/ery164)
Supplement: Supplementary Figures and Tables [file ery164_suppl_supplementary_figures_tables.pdf]

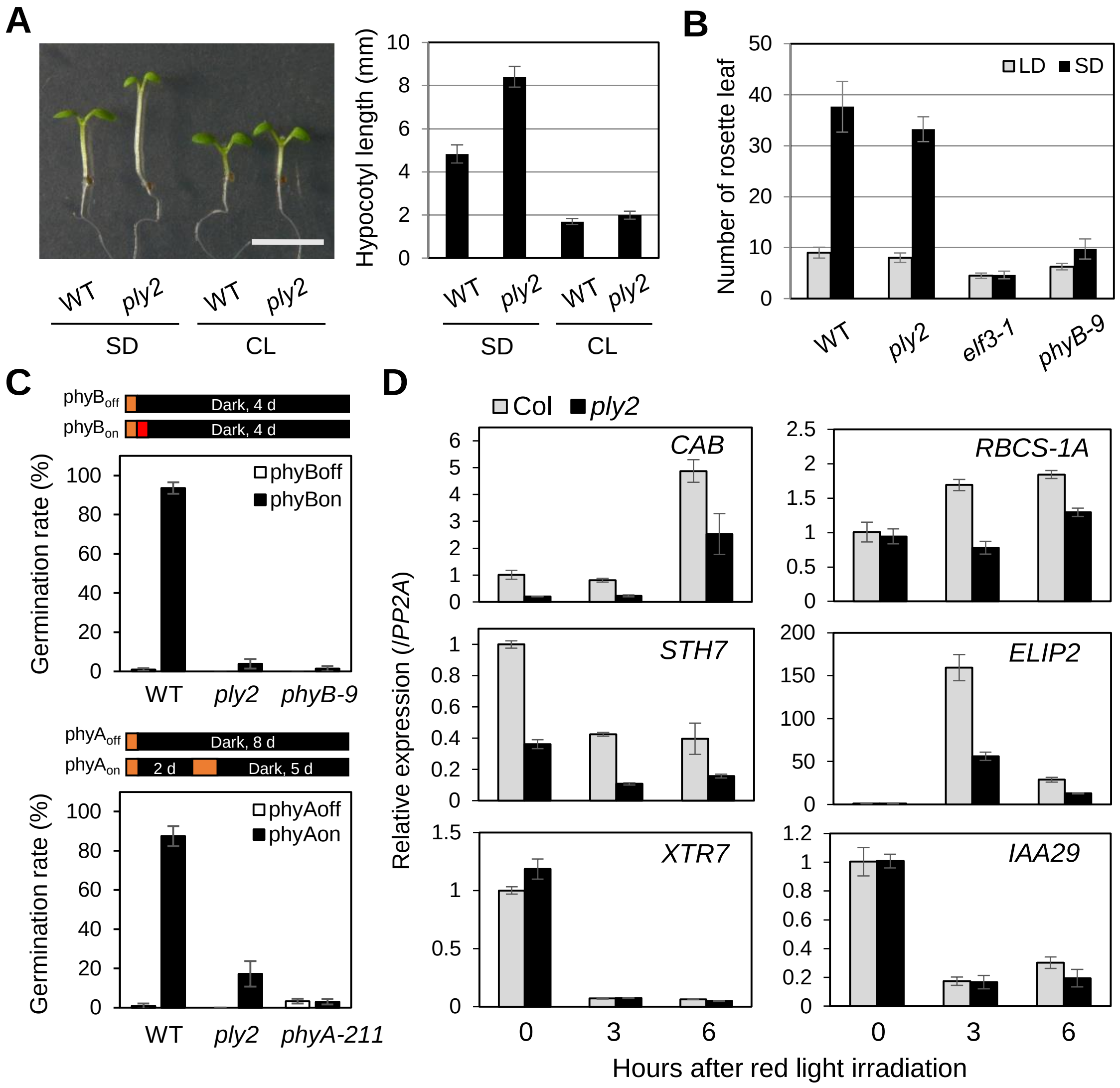

Fig. S1. Identification of *ply2* mutant with long hypocotyl phenotype. **(A)** Wild type (Col) and *ply2* seedlings were grown under either short-day (SD) conditions (10 h L/14 h D) or continuous light (CL) for 5.5 days. Left, phenotypes of representative seedlings. Scale bar indicates 5 mm. Right, The shown are average length of hypocotyls from at least 15 seedlings (n=15-17). Error bars, SDs. **(B)** Flowering phenotype of *ply2* mutant. Flowering time was measured as average number of rosette leaves at bolting time. Plants were grown under long-day (LD, 16h L/8h D) or short-day (SD, 10h L/14h D) condition (n=12). Error bars, SDs. **(C)** Phenotypes of *ply2* mutant during phytochrome-dependent germination. Upper; Germination rate of *ply2* mutant seeds were examined under phyB-dependent germination condition. The *phyB-9* seeds were used as experimental control. Lower; Germination rate of *ply2* mutant seeds were examined under phyA-dependent germination condition. The *phyA-211* seeds were used as experimental control. (D) Altered light-responsive gene expression of *ply2* mutant. The 4-days-old dark-grown seedlings were given with red light (5.72 μW cm^{-2}) for indicated hours before extraction of total RNA. The graph shown are average values of relative expression level of each gene tested after normalization with the transcript level of *PP2A*, in which the level of dark-grown wild type was set to 1. Error bars, SDs from experimental replicates (n=3).

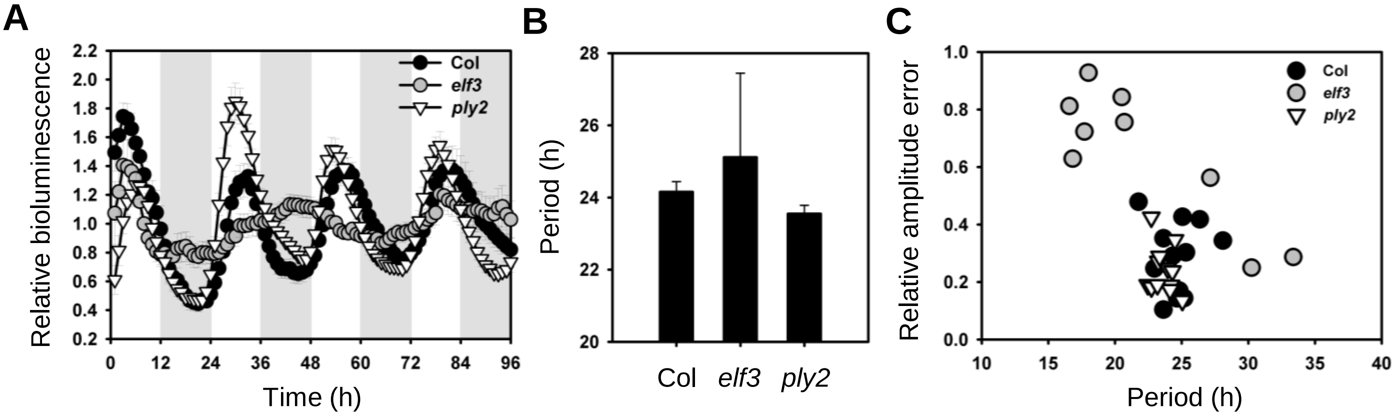

Fig. S2. Circadian rhythm phenotype of *ply2* mutant. **(A)** Bioluminescence traces from *CAB2:LUC* in LL. **(B and C)** Mean period (B) and Relative amplitude error (RAE) (C) in wild type (Col), *elf3-1* mutant (*elf3*) and *ply2* mutant. Plants were grown under 12L:12D for 7 days and transferred to continuous white light. Luminescence intensities were measured every hour and were normalized to the mean expression level over the 0 to 96 h sampling schedule (mean ± SE). Period and RAE were analyzed by FFT-NLLS using bioluminescence data obtained from 24 to 96 h and period lengths are shown as partially variance-weighted periods ± SE.

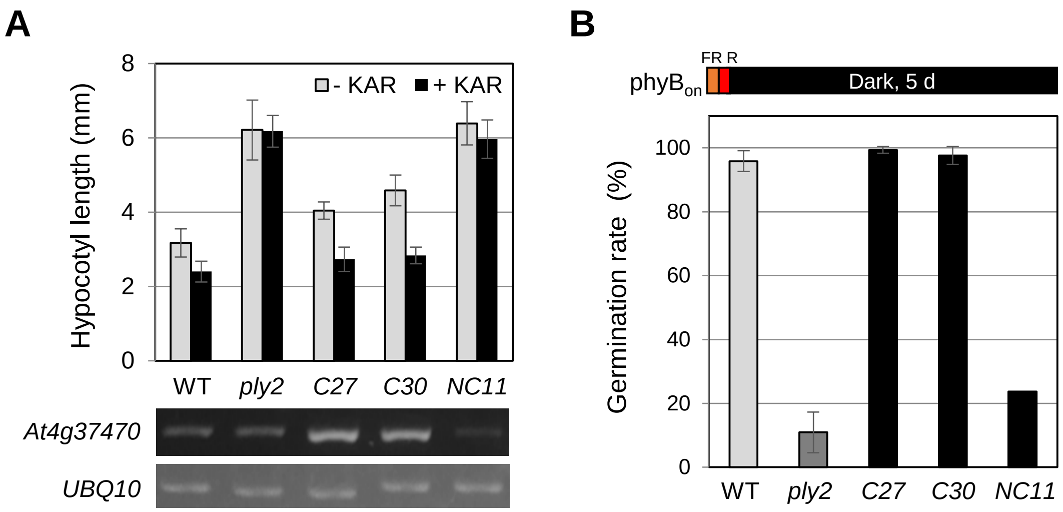

Fig. S3. Transgenic complementation of *ply2* mutant. **(A)** Overexpression of *At4g37470* restored altered hypocotyl growth and response to KAR in *ply2*. The seedlings were grown under short day light condition (10 h L/14 h D) for 5 days on MS media with or without 10 μM KAR_2. Average length of hypocotyl were shown along with SD. Lower, RT-PCR analysis of the expression level of *At4g37470* in transgenic lines tested. Total RNA was extracted from 6 days-old seedlings which were grown under continuous light. *UBQ10* was used as internal control. **(B)** Overexpression of *At4g37470* restored reduced phyB-dependent germination of *ply2.* After sterilization, seeds of the wild type and the mutants were placed aqueous medium, then irradiated with far-red light (1.325 μW cm^{-2}) for 15 min with subsequent red light (5.72 μW cm^{-2}) for 10 min ($phyB_{on}$). After incubation for 5 days under dark, germination rate was measured. *C27* and *C30* are independent *KAI2*-overexpressing transgenic lines, complementing *ply2* mutant phenotypes, and *NC11* is a non-complementing transgenic line. Average of germination rates from experimental replicates were shown along with SD (n=3).

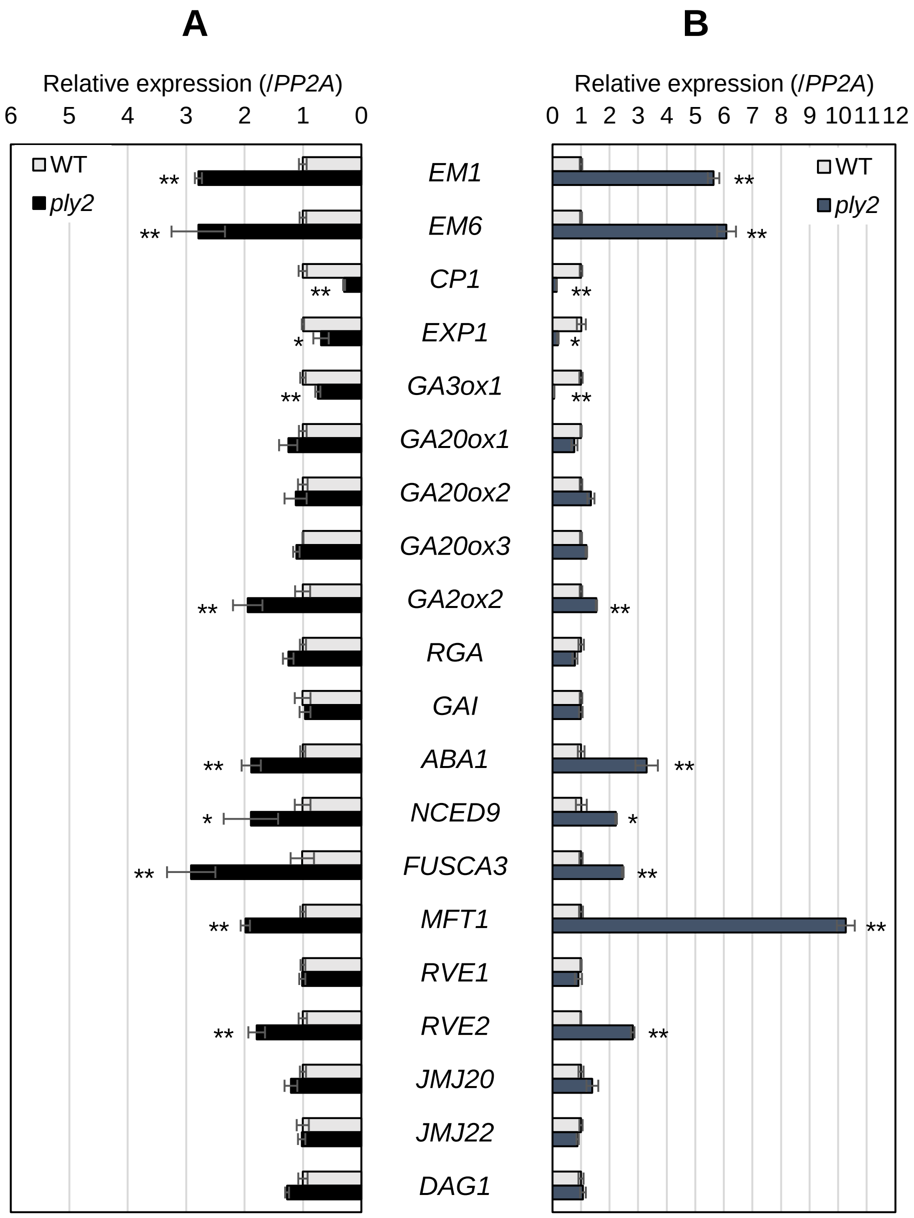

Fig. S4. Additional biological repetitions of gene expression profiling under phyB-dependent germination condition. Quantitative real-time PCR analysis was performed as in Fig. 2A with two independent seed batches for (A) and (B). Total RNA was extracted from 12 h-imbibed seeds under $phyB_{on}$ condition. The graphs shown are average values of relative expression of genes after normalization with the transcript level of *PP2A*. Error bars indicate standard deviations from experimental replicates (n=3). Asterisks denote statistically significant difference from the expression level of wild-type, analyzed by student t-test (**, $p < 0.01$; *, $p < 0.05$).

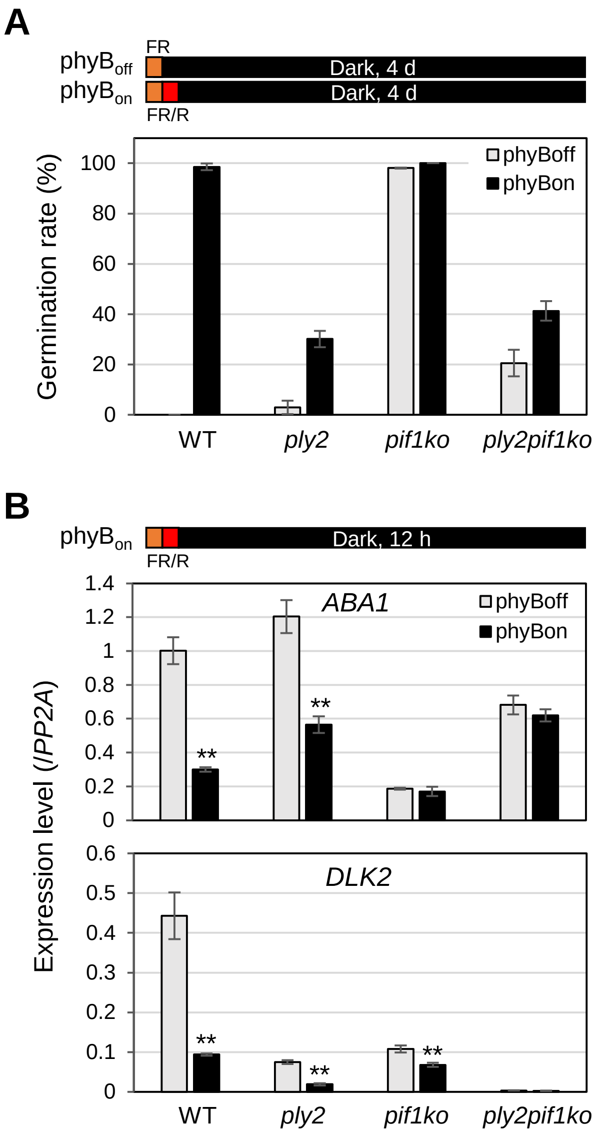

Fig. S5. Double mutant analysis between *ply2* and *pif1* mutants. **(A)** PhyB-dependent germination assay. Seeds were surface-sterilized and sown onto aqueous media (pH, 5.8). After irradiation with far-red (FR) light (1.325 μW cm^{-2}) for 10 min, the plates were treated with red (R) light (5.72 μW cm^{-2}) for 10 min. Then the plates were wrapped with layers of aluminum foil and kept in darkness for 4 days. Germination rates were calculated with experimental replicates (n=3) with at least 50 seeds per plate and averaged. Error bars, SDs. **(B)** Quantitative real-time PCR analysis. Total RNA was extracted from 12 h-imbibed seeds under $phyB_{on}$ or $phyB_{off}$ condition. The graphs shown are average values of relative expression of *ABA1* and *DLK2* genes after normalization with the transcript level of *PP2A*. Error bars indicate standard deviations from experimental replicates (n=3). Asterisks denote statistically significant difference from the expression level under $phyB_{off}$ condition, analyzed by student t-test. **, $p < 0.01$.

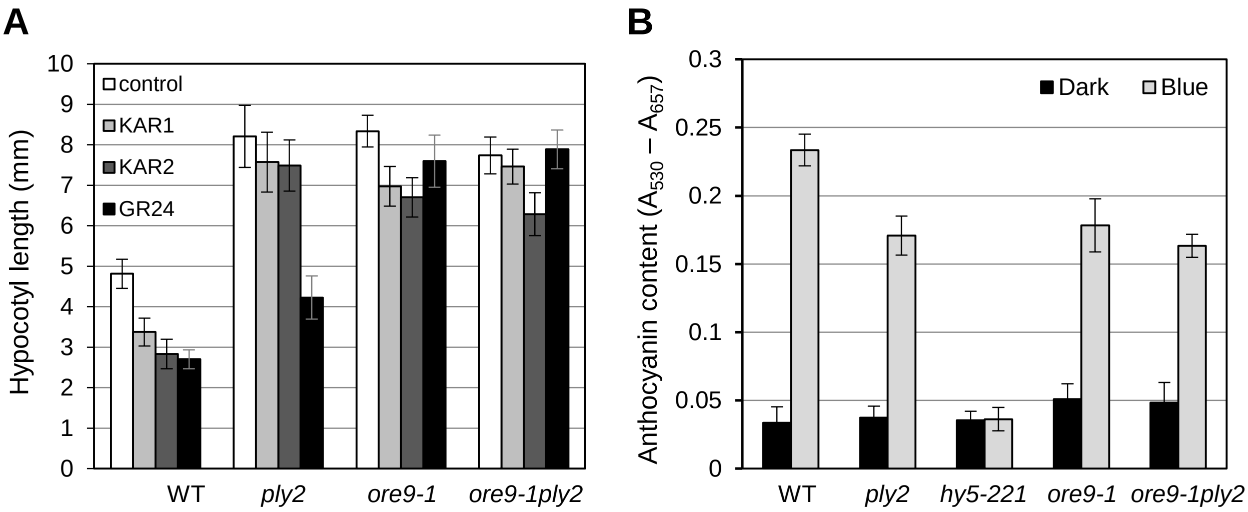

Fig. S6. Double mutant analysis between *ply2* and *ore9-1* mutants. The *ply2* mutant phenotypes were not further enhanced by *ore9-1* mutation. **(A)** Karrkin- and GR24-responsive hypocotyl growth. The seedlings were grown on MS-Suc (1%) in short day light condition (10 h L/14 h D) for 5 d with or without 10 μM of $karrikin_1$ (KAR_1), $karrikin_2$ (KAR_2), and *rac*-GR24 (GR24) respectively. The shown are average length of hypocotyls from at least 15 seedlings (n=15-18). Error bar indicates standard deviation (SD). **(B)** Light-dependent anthocyanin accumulation. Extraction of anthocyanin was performed with seedlings that were grown on MS-Suc (1%) for 3 d under blue light (17.4 μW cm^{-2}) or in darkness after irradiation with white light for 12 h. Average values were obtained from experimental replicates (n=3). Error bars, SDs.

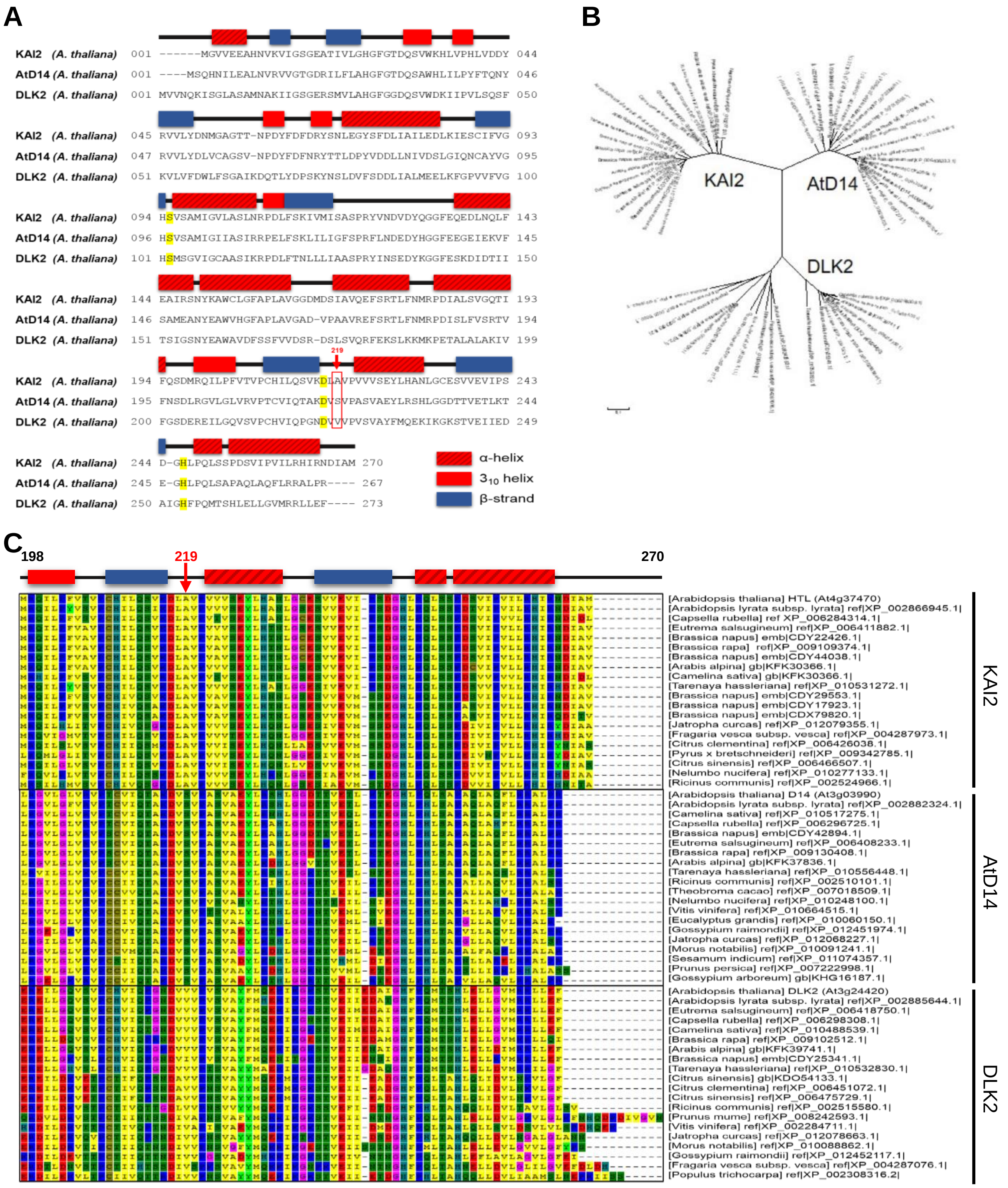

Fig. S7. Phylogenetic analysis of KAI2 homologs. (A) Sequence alignment of KAI2, AtD14, and DLK2 proteins of *Arabidopsis thaliana*. The secondary structures of KAI2 are shown at the top. The catalytic triad (Ser95, Asp217 and His246) residues (yellow) and the mutated residue, Ala219 of KAI2 in *ply2* mutant (red) are highlighted. (B) Phylogenetic analysis of KAI2 homologs found in several plants. Phylogenetic analyses were conducted in MEGA6. The evolutionary history was inferred by using the Maximum Likelihood method. (C) Multiple sequence alignment of the KAI2, AtD14, and DLK2 homologs. Numbers indicate the amino acid position of KAI2. Secondary structures of KAI2 are shown at the top. The red arrow denotes Ala219 of KAI2, the mutated residue in the *ply2* allele.

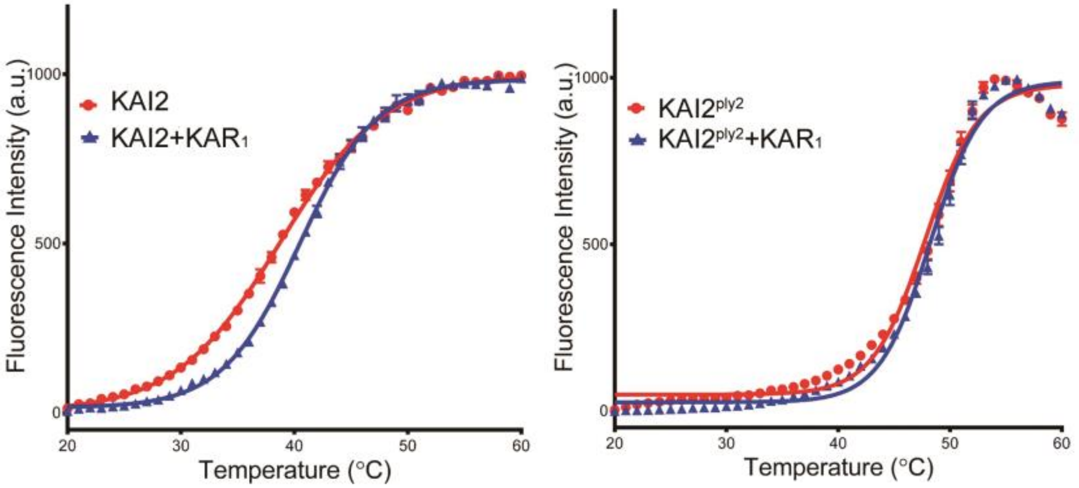

Fig. S8. CPM assay of KAI2 and KAI2^{ply2}. For CPM assay, the recombinant KAI2 and KAI2^{ply2} proteins were used, adjusted to final concentration of 51 μM. For karrikin-binding assay, 1,000 μM of KAR_1 was added before the thermal denaturation assay. The shown are average values from experimental triplicates, along with standard deviations. SigmaPlot was used for curve-fitting.

Supplementary Table S1. Thermodynamic properties determined by ITC.

	Kd (μM)	*ΔH* (kcal mol^{-1})	*ΔS* (kcal mol^{-1} deg^{-1})	*ΔG* (kcal mol^{-1})
KAI2	147.5	-5.67	-1.47	-4.20
$KAI2^{ply2}$	2,816.9	-51.71	-0.16	-51.55

Supplementary Table S2. Statistics of X-ray data collection and refinement.
* PDB, Protein Data Bank, *r.m.s.*, root mean squared.
† Highest-resolution shell is shown in parenthesis.

PDB	KAI2(5Z9G*)	KAI2^{ply2}(5Z9H*)
Data Collection		
Space group	P1211	P1211
a, b, c (Å)	50.60, 55.78, 53.03	50.84, 55.49, 53.02
α, β, γ (˚)	90.00, 116.07, 90.00	90.00, 116.42, 90.00
Resolution (Å)	50.00 – 1.49<br>(1.52 – 1.49)†	50.00 – 1.49<br>(1.52 – 1.49) †
Rmerge (%)	6.8 (16.1)†	5.6 (16.5)†
I/σ	54.1 (14.4)†	48.8 (10.1)†
Completeness (%)	99.7 (99.4)†	99.6 (98.6)†
Redundancy	6.1 (5.2)†	6.4 (5.4)†
Refinement		
Resolution (Å)	27.89 – 1.49	27.75 – 1.49
No. reflections	43,333	43,060
Rwork/Rfree (%)	13.78/17.49	13.41/16.80
No. atoms	2,295	2,271
Protein	2,066	2,073
Water	229	198
B factors (Å^2)		
Protein	14.80	12.20
Water	28.50	23.70
r.m.s. deviations		
Bond lengths (Å)	0.005	0.006
Bond angles (˚)	1.04	1.03

Supplementary Table S3. Primers used in this study.

Primers for chromosomal mapping

Marker name	Nearby AGI	Marker type	Forward primer (5'-3')	Reverse primer (5'-3')	Product size
F15J1-1	AT4G35480	SSLP	AATTCAAGTTTGATGTTCTAC	AAAGTTCAAAGCATGCATTA	Col : 122bp / Ler : 104bp
AP21-1	AT4G37295	SSLP	TTGTGCAATATATAATGTTC	AATGATGGACATGCAAGAT	Col : 129bp / Ler : 117bp
F6G17-1	AT4G37400	SSLP	AGTTCTGATCTCCTCCCATC	TGCCAGGTAATTGCATGTGA	Col : 160bp / Ler : 147bp
F6G17-SspI	AT4G37490	CAPS	CTGTGCTGGGGTGTTTGAT	TGATTCATTCCCAAACACTGCA	SspI digestion,<br>Col : 88 + 75bp / Ler : 163bp
F19F18-1	AT4G37580	SSLP	TGACGGTTGATATTTCGGCT	CCTCTTTTAAGGGTAGTTTAGTC	Col : 187bp / Ler : 179bp
T28I19-1	AT4G37760	SSLP	CATATCAATCAATCGAATTA	AACAGAACTGGATGAAGTTA	Col : 130bp / Ler : 120bp
nga1107	AT4G38770	SSLP	GCGAAAAAACAAAAAAATCCA	CGACGAATCGACAGAATTAGG	Col : 150bp / Ler : 140bp

Primers for plasmid constructs

Primer name	Sequence (5'-3')
pENT-at4g37470-F	GGTACCGAAGAATCTTCTTTAGAGAGATG
pENT-at4g37470-XR	CTCGAGTCCATAGCAATGTCATTACGAA

Primers for genotyping

Mutant name	Mutant ID	Forward primer (5'-3')	Reverse primer (5'-3')
pif1-ko	SALK_072677	GGTACCATGGATCCTCAGCAGCAACC	GAATTCGCTCTTTGACACTACAGATG
smax1-3	SALK_097346C	AGCAGGCCAAGAAGAAGAGTCC	TACTGCCAAAGTAATAGTTGTCGG
Salk left border (Ecker LBaI-2)		TGATGGTTCACGTAGTGGGCCATCG	
*ore9-1**		AGTTAAGAGTATTGAAGCTAGTA	CGTAGTCAACTTACAACATCCT
*ply2***		CTCCAAAGTGTTAAGGACTCAG	GACTAGACAGACATATCACATC

* *ore9-1* mutant was confirmed by digestion of PCR product with RsaI. (Col : 154 bp / *ore9-1* : 176 bp)

** *ply2* mutant was confirmed by digestion of PCR product with PvuII. (Col : 241 bp / *ply2* : 263 bp)

Primers for RT-PCR

Gene name	Forward primer (5'-3')	Reverse primer (5'-3')
UBQ10	GATCTTTGCCGGAAAACAATTGGAGGATGGT	CGACTTGTCATTAGAAAGAAAGAGATAACAGG
At4g37470	CGTCGTCCTCTACGACAACATGG	ATGTCGGGACGCATATTGAAGAGTG

Supplementary Table S3. Primers used in this study (continued).

Primers for qRT-PCR

Gene name	Gene ID	Forward primer (5'-3')	Reverse primer (5'-3')
PP2A	*AT1G13320*	TATCGGATGACGATTCTTCGTGCAG	GCTTGGTCGACTATCGGAATGAGAG
CAB1	*AT1G29930*	CCCATTTCTTGGCTTACAACAAC	TCGGGGTCAGCTGAAAGTCCG
RBCS1A	*AT1G67090*	CAGTCACACAAAGAGTAAAGAAG	CTTAGCCAATTCGGAATCGGT
STH7	*AT4G39070*	CATCTCCGGTTCTCTCTCACTTCT	CATTCTCTGCATAGTATTGCTCTGTC
ELIP2	*AT4G14690*	GATCCTTCTGTGCCCTCGACCTC	GGTGAAGGCCAACGCAACGAGAC
XTR7	*AT4G14130*	CGGCACCGTCACTGCTTAC	GAAACTCAAAGTCTATCTCGTCATGTG
IAA29	*AT4G32280*	TCATCATCGTGGTAGGAGGATC	GTATATGCACACGGTCGATCTC
DLK2	*AT3G24420*	GTTTTGGTCTTTGACTGGCTTT	TTTGAAGGGTCATAGAGAGTTTGA
KUF1	*AT1G31350*	AACCCGTCAGTCCGGTTTATGTG	AACGACGGATGACGGTAAAGAATCC
At3g60290	*AT3G60290*	TGGCTCGCTGACAATCTTACTCC	CGGCACACAAACCCAGTTGTTG
EM1	*AT3G51810*	CGGAGGAAGAAGGGATTGAGA	TGCCAAACACGGAACCTACA
EM6	*AT2G40170*	AGGATATCAGCAGATGGGACGC	CGTCTATCTCGACTCCTTCCTC
CP1	*AT4G36880*	AACGGCGGTTTAATGGACTACGC	GCTTTCTTCAACGCAGTCTCGTCTT
EXP1	*AT1G69530*	CTGGATGGCAAGCGATGTCAAGA	TCATGCCCCTCTCTCTAACTGCTTC
GA3ox1	*AT1G15550*	GGGTGCCTTCCAAATCTCAAACC	TCGCTGACCCCAAGTGAATTTAGTG
GA20ox1	*AT4G25420*	CCGTTCAAGATTACTTCTGCGATGC	AGCGCCATTGATTTTCCACAAAGAC
GA20ox2	*AT5G51810*	CACCGGCAGATTCTCCACTAAGC	TTGGCATGGAGGATAATGATTGAGC
GA20ox3	*AT5G07200*	AGACAGCGATTCAATATTCCGGTTG	CTGGTGGCTTCACCACTTTTTCC
GA2ox2	*AT1G30040*	CAGGGTCTTAGCCGATACAAGGAG	GTGCGATCTTCTGGCTCAATGG
RGA	*AT2G01570*	CATTCCCGGAAACGCGATTTATCAG	TCACCGTCGTTCCTATGACTCCA
GAI	*AT1G14920*	AGCGTCATGAAACGTTGAGTCAGTG	TGCCAACCCAACATGAGACAGC
ABA1	*AT5G67030*	GATGCAGCCAAATATGGGTCAAGG	GCCATTGCATGGATAATAGCGACTC
NCED9	*AT1G78390*	GGAAAACGCCATGATCTCACA	GCAGGATCCGCCGTTTTAG
FUSCA3	*AT3G26790*	TGTGAATGCTCATGGTCTGC	GGAGGAGAAGATCGTTAACCAC
MFT1	*AT1G18100*	ATCACTAACGGCTGCGAGAT	CGGGAATATCCACGACAATC
RVE1	*AT5G17300*	CTCCTCGTCCCAAGAGAAAG	GTGGACAACACAGAGGTTGG
RVE2	*AT5G37260*	CTAACCGGATCCAAGCTGAT	GAACCTAATCCATCTGAGCCA
JMJ20	*AT5G63080*	TCCCAGTGGATGGCATCATCAAG	TCCTTCCACAGTAGATCCCACACC
JMJ22	*AT5G06550*	TGTTCCCACCTGATGTGGTTCC	TGGAAACAGGACAGGCCACTTC
DAG1	*AT3G61850*	TTGTCGAAGGTATTGGACCGA	CCGACTGGGACGTTACGAAG
KAI2	*AT4G37470*	TGGCTTCTCTTAACCGTCCT	CTTCGGATGGCTTCGAATAGT
